# Dynamic Loading and Tendon Healing Affect Multiscale Tendon Properties and ECM Stress Transmission

**DOI:** 10.1038/s41598-018-29060-y

**Published:** 2018-07-18

**Authors:** Benjamin R. Freedman, Ashley B. Rodriguez, Ryan J. Leiphart, Joseph B. Newton, Ehsan Ban, Joseph J. Sarver, Robert L. Mauck, Vivek B. Shenoy, Louis J. Soslowsky

**Affiliations:** 1 McKay Orthopedic Research Laboratory, University of Pennsylvania, Philadelphia, PA USA; 2 Department of Bioengineering, School of Engineering and Applied Science, University of Pennsylvania, Philadelphia, PA USA; 3 Department of Materials Science and Engineering, School of Engineering and Applied Science, University of Pennsylvania, Philadelphia, PA USA; 4 Department of Biomedical Engineering, Drexel University, Philadelphia, PA USA; 5 John A. Paulson School of Engineering and Applied Sciences, Harvard University, Cambridge, MA USA; 6 Wyss Institute for Biologically Inspired Engineering, Harvard University, Boston, MA USA; 7 Center for Engineering Mechanobiology, University of Pennsylvania, Philadelphia, PA USA

## Abstract

The extracellular matrix (ECM) is the primary biomechanical environment that interacts with tendon cells (tenocytes). Stresses applied via muscle contraction during skeletal movement transfer across structural hierarchies to the tenocyte nucleus in native uninjured tendons. Alterations to ECM structural and mechanical properties due to mechanical loading and tissue healing may affect this multiscale strain transfer and stress transmission through the ECM. This study explores the interface between dynamic loading and tendon healing across multiple length scales using living tendon explants. Results show that macroscale mechanical and structural properties are inferior following high magnitude dynamic loading (fatigue) in uninjured living tendon and that these effects propagate to the microscale. Although similar macroscale mechanical effects of dynamic loading are present in healing tendon compared to uninjured tendon, the microscale properties differed greatly during early healing. Regression analysis identified several variables (collagen and nuclear disorganization, cellularity, and F-actin) that directly predict nuclear deformation under loading. Finite element modeling predicted deficits in ECM stress transmission following fatigue loading and during healing. Together, this work identifies the multiscale response of tendon to dynamic loading and healing, and provides new insight into microenvironmental features that tenocytes may experience following injury and after cell delivery therapies.

## Introduction

**Figure 1:**
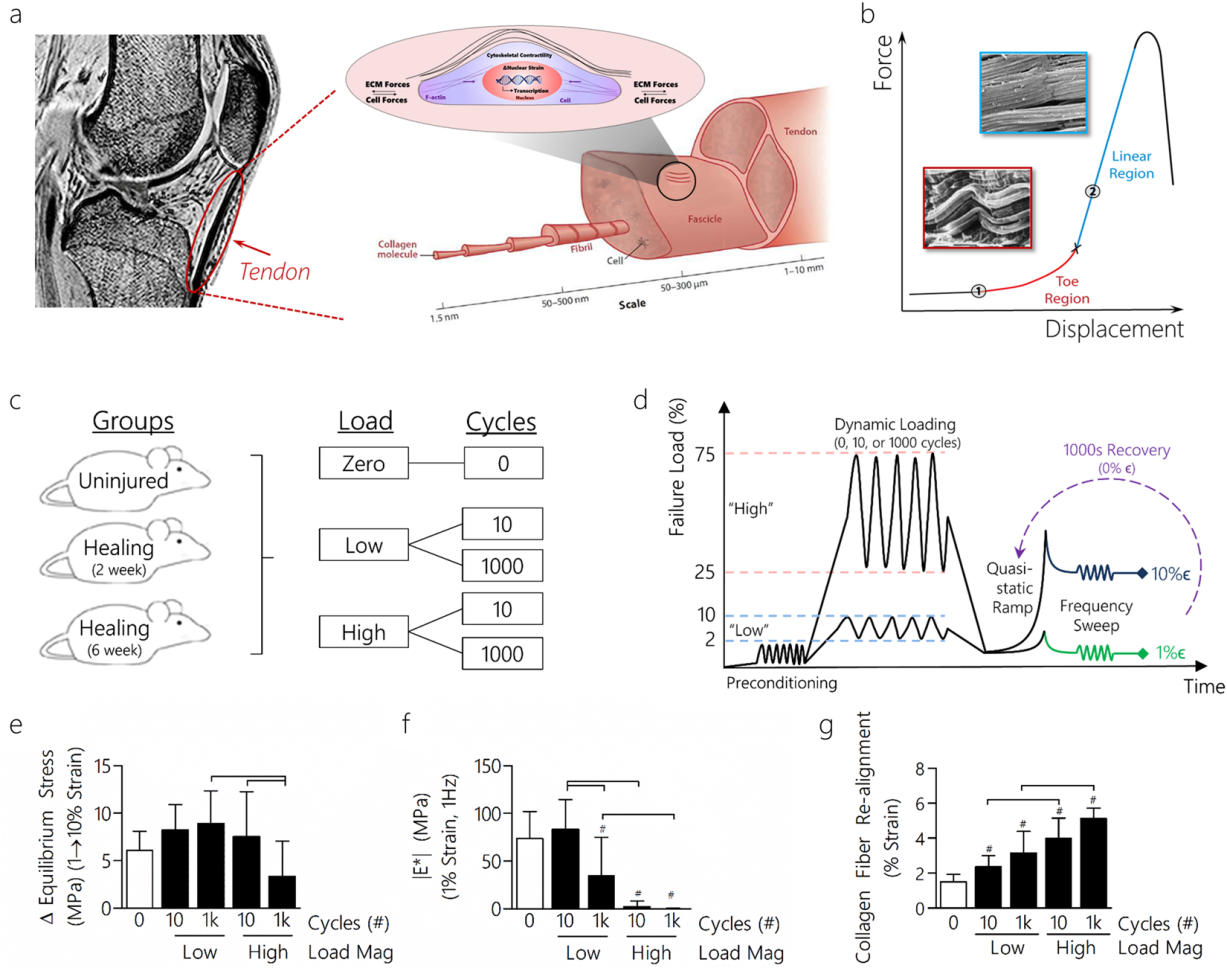
Magnitude and duration of dynamic loading affects macroscale tendon properties. (**a**) Tendon, which connects muscle to bone, has a hierarchical structure that spans several length scales and comprises both a collagenous matrix and cellular components. Tendon cells maintain tensional homeostasis by balancing cell and ECM forces. (**b**) Force and displacement have a nonlinear relationship during tensile loading as disorganized fibers operative in the toe region become organized in the linear region. (**c**) Tendon groups were loaded at different load levels (zero, low, high) and cycle durations (0, 10, 1000) prior to multiscale property evaluation. (**d**) Tendons were preconditioned and then dynamically loaded for 0, 10, or 1000 cycles at either high (25–75% UTS) or low (2–10% UTS) loads. Following loading, tendons underwent a quasi-static ramp to 1% or 10% strain followed by a frequency sweep. For recovery experiments, tendons were allowed 1000 s of rest at 0% strain prior to a second quasi-static ramp. For non-recovery experiments, tendons were snap frozen at either 1% or 10% strain for microscale assessment. (**e**) The change in equilibrium stress was decreased following high magnitude long duration loading. (**f**) The dynamic modulus, |E*|, also decreased following long duration and high magnitude loading. (**g**) The strain at which collagen fiber re-alignment occurred was elevated due to long duration and high magnitude loading. Data shown as mean ± SD. N = 7–11/group. Lines indicate significant differences. Symbols indicate significant differences to quasi-static controls (unshaded).

Tendons are dense fibrous connective tissues that transmit forces and displacements between muscles and bones to stabilize joints and generate skeletal movement (Fig. 1a). During macroscale tensile loading, tendons strain stiffen, as is evidenced by the distinct nonlinearity, or toe region, in the load-displacement curve that becomes linear with increased displacement prior to ultimate failure (Fig. 1b)^1,2^. Loading induced changes to the extracellular matrix (ECM) of tendon occur across several length scales (tendon, fascicle, and fibril levels), and give rise to dynamic processes such as fiber uncrimping and realignment^1^. Together, disorganized and crimped fibers in the toe region organize and uncrimp as they enter the linear region. The fiber-reinforced structure of the tendon allows strain transfer not only between ECM components, but also from the ECM to tendon cells (e.g., tenocytes). Indeed, applied tissue strains correlate to cell strains in uninjured fibrous tissues^5^.

Several clinically relevant scenarios may affect strain transfer in tendon. When tendons are subjected to high magnitude cyclic loading (fatigue loading), a 3-phase pattern in macroscale strain-cycle response is observed prior to tissue-level failure^6–8^, together with emergent domains of collagen fiber kinking^9^ and cell rounding at the microscale^10^. After injury, “healed” tendon remains biologically, structurally, and mechanically inferior to native tissue and can exhibit non-tendon-like phenotypes, including bone formation or heterotopic ossification, deposits of cartilage, and rounded cell shape and high cell numbers^11,12^. In neighboring regions of fibrous components in tissues such as the meniscus, the presence of such disordered inclusions resulted in reduced strain transfer to the endogenous cells and altered their mechanosensing and response^5,13^.

The ability of tendon to maintain its homeostatic state following dynamic loading and return to a native condition after injury is governed in part by the restoration of native multiscale strain transfer mechanisms. As forces through the extracellular matrix are applied, cell deformation through the actin cytoskeleton to the nucleus induces nuclear strain that, in turn, can affect transcription and a host of cell responses, such as inflammation, migration, proliferation, and differentiation^3,4,14,15^. Although mechanical loading can affect gene and protein expression^16–20^, explication of how applied strains regulate the nuclear shape changes that drive these downstream responses remains limited. The ability or hindrance of cells to deform under applied strain may have important physiological consequences and may provide a therapeutic target. Forces transferred from the ECM to cells may be balanced by traction forces exerted by the cell during cytoskeletal contraction leading to ECM stress transmission^21^. ECM stress transmission has important implications, including cell-cell communication, and can drive tissue patterning and re-arrangement^21–24^. Changes in ECM stress transmission may provide feedback to promote a healthy (e.g., highly aligned collagen and spindle-like cells) or a pathologic matrix phenotype (Figure S1).

The overall objective of this study was to investigate the role of mechanical loading (quasi-static and dynamic) and tendon healing on multiscale mechanical, structural, and compositional properties. In addition, we developed computational models to predict nuclear shape and ECM stress transmission. We hypothesized that dynamic loading and healing would affect multiscale matrix and cell properties that can either promote or impede changes in nuclear shape and ECM stress transmission.

## Results

### Macroscale Mechanics and Structure are Compromised After High Load Magnitude Dynamic Loading in Uninjured Living Tendon

We first investigated how macroscale mechanical properties vary as a consequence of dynamic loading by quantifying changes in tissue strain stiffening in uninjured tendon (Fig. 1c). We hypothesized that loading magnitude (to 10% (‘low’) or 75% (‘high’) of the ultimate tensile strength) and duration (0, 10 or 1000 cycles) would affect macroscale strain stiffening (Figure S2). After loading, we evaluated strain stiffening by loading tendons to either 1 or 10% strain followed by stress relaxation and a frequency sweep (Fig. 1d). We also applied a fiber recruitment model to determine the mean slack length at which fibers uncrimp^25^ after these various mechanical perturbations.

In agreement with our hypothesis, cycle number and loading magnitude affected tissue strain stiffening. That is, the equilibrium stress decreased in the high/1k cycle group (high/1k denotes high magnitude loading for 1000 cycles) compared to the high/10 and low/1k cycle groups (Fig. 1e). This decrease in strain stiffening was coupled with a decreased dynamic modulus in the longer duration and high magnitude loading groups at 1% strain (Fig. 1f). The dynamic modulus was decreased in the high/1k group at 10% strain (Figure S4d). Cycle number was a significant factor, regardless of applied load, on increasing tendon laxity (Figure S4a). As tendons were loaded dynamically in different regimes of the stress-strain curve, the secant modulus showed a significant increase in the high magnitude loading cases, as expected (Figure S4b). In agreement with decreased strain stiffening and increased laxity, elevated fiber slack lengths were predicted in the high/1k group (Figure S4c).

We next verified our predictions of increased slack lengths due to high magnitude loading by assessing collagen fiber re-alignment. Macroscale collagen organization was measured using polarized light imaging. A crossed polarizer system was integrated with a mechanical testing setup to nondestructively assess collagen fiber alignment during loading. Using this approach, we found that the low/10 loading group resulted in more collagen fiber re-alignment at lower strains compared to the high/1k loading group (Fig. 1g). Taken together, these results suggest that tendon strain stiffening is reduced as a consequence of high magnitude and longer duration loading, and that this occurs in concert with increased laxity and delayed fiber re-alignment with applied strain. Notably, these macroscale mechanical property changes with high dynamic loading were non-recoverable after 1000 s of rest at 0% strain, indicating that permanent macroscale mechanical alteration was present in these groups and that this loading protocol was indeed fatigue loading the tendons (Figure S4e,f).

### Microscale Structural and Compositional Properties are Compromised Following High Load Magnitude Dynamic Loading

**Figure 2:**
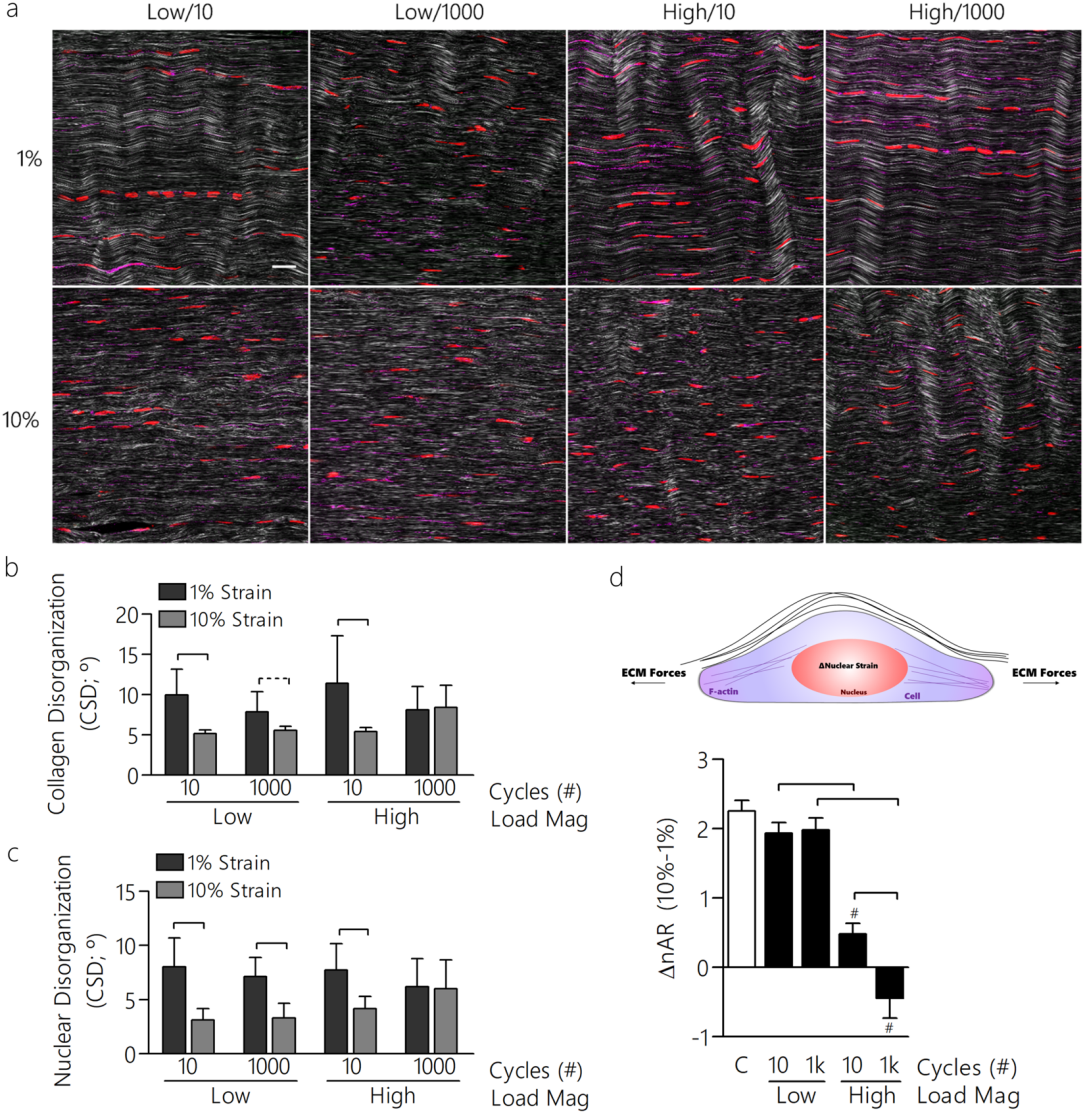
Microscale tendon properties are altered with dynamic loading. (**a**) Multiphoton imaging was used to quantify collagen organization (white), amount of F-actin (purple), and nuclear aspect ratio (red) and disorganization (scale bar = 20 µm). (**b**) Collagen disorganization decreased with applied strain in all groups except those that had been subjected to high/1k loading, similar to (**c**) the nuclear disorganization. (**d**) The change in nuclear aspect ratio (ΔnAR) with applied strain also decreased in high magnitude loading groups. *Panels a–c: Data shown as mean ± SD. N = 7–11/group. Lines indicate significant differences. *Panel d: Data shown as mean ± SEM. N = 119–815 cells/group.

Several *in vitro* bioreactor studies have evaluated the effects of cyclic loading on tendon macromechanics^16,17,26–29^, inflammatory cytokines^26,30^, ECM components^17,30–32^, and gene expression^16,18,33,34^. However, none of these studies examined potential acute changes to the tendon cell microenvironment and resulting morphological properties of the nucleus or surrounding matrix that may play important roles in mechanotransduction. To investigate whether macroscale changes in mechanics and structure propagated to the microscale, we next evaluated tendon structure using multiphoton (MP) imaging and simultaneously evaluated tendon cell nuclear shape and F-actin morphology by varying the magnitude and duration of mechanical loading (Fig. 2a). We hypothesized that microscale collagen disorganization, nuclear shape, and nuclear disorganization would be altered and less responsive to applied strain in tissues that had been conditioned under high load dynamic loading compared to low load dynamic loading. Following mechanical loading, tendons were maintained at 1 or 10% strain using a snap freezing process prior to cryosectioning and MP imaging to assess nuclear shape, F-Actin, and collagen organization.

Importantly, we used living tendon explants that preserve the native architecture, biology, and structure of the ECM in tendon. Due to the slow timescale of matrix remodeling in tendon^35^, it is likely that alterations in matrix properties (e.g., mechanics and structure) precede cell responses. The methods used are necessary for validation in tissue engineered constructs that may over estimate strain transfer compared to native tissue^5^. This remains critical for accurate comparisons to model *in vivo* microenvironments. Following sacrifice, tendons were quickly dissected while hydration was maintained prior to mechanical testing in a bioreactor-like setup integrated with a mechanical testing device. We first confirmed that cell viability was maintained during the 25-min mechanical test using an MTT assay (Figure S3). To quantify organization of collagen, MP imaging was performed. Using images obtained with forward scatter, we developed a fiber organization algorithm to quantify fiber angles throughout the ROI (Figure S4). This dispersion of fiber angles is represented as the circular standard deviation (CSD). A larger CSD indicates increased fiber disorganization. To probe cellular features in this fibrous network, nuclear morphologies were quantified following segmentation (CellProfiler^36^) by computing the nuclear aspect ratio (ratio of long and short axes) and average disorganization (Figure S6). Confocal and MP imaging revealed changes in collagen organization, nuclei shape, and nuclei organization that were dependent on the loading protocol and applied strain (Fig. 2b). Although all groups showed similar collagen organization regardless of loading protocol at 1% strain, fatigue loaded samples showed increased fiber disorganization at 10% strain, and thus were strain insensitive. Similar negative effects of fatigue loading resulted in a reduced strain transfer to nuclei; nAR and ΔnAR from 1% to 10% strain was diminished in fatigue loaded samples compared to low-magnitude loading and quasi-static controls (Fig. 2d, S7). This decreased ΔnAR was coupled with increased nuclei disorganization in the high/10 and high/1k loaded tendons (Fig. 2c), that remained strain insensitive following fatigue loading. Nuclear disorganization correlated linearly with collagen disorganization (R^2^ = 0.90).

### Dynamic Loading Affects Macroscale Tendon Properties in Healing Tendon

**Figure 3:**
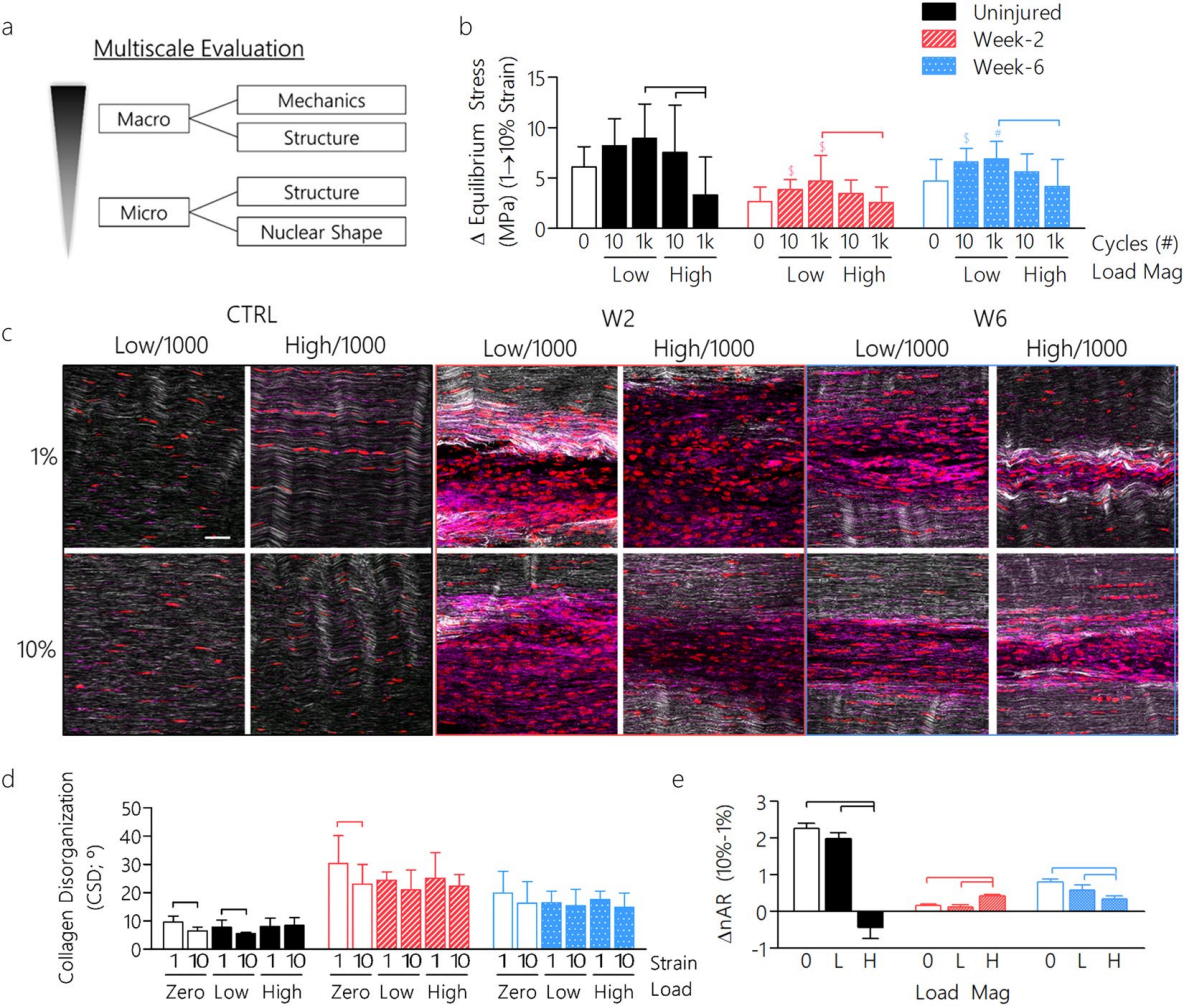
Multiscale tendon properties are altered with dynamic loading during tendon healing. **(a)** Healing tendons were evaluated for multiscale properties (macro and micro-scale) following dynamic loading. **(b)** Similar to uninjured tendon, the change in equilibrium stress was decreased in healing tendons due to high magnitude long duration loading. (**c**) Multiphoton imaging was used to assess collagen organization (white), amount of F-actin (purple), and nuclear aspect ratio (red) and disorganization following dynamic loading at different stages of healing (scale bar = 20 µm). (**d)** Collagen disorganization was strain responsive with dynamic loading in uninjured tendons, but was not responsive in healing tendons. (**e**) The change in nuclear aspect ratio decreased following high magnitude dynamic loading in uninjured and week-6 post-injury groups, but increased in the week-2 post-injury healing groups. *Panels b,d: Data shown as mean ± SD. N = 7–11/group. Lines indicate significant differences. Symbols indicate significant differences (#) or trends ($) compared to quasi-static loading samples (0 cycles). *Panel e: Data shown as mean ± SEM. N = 119–1012 cells/group.

Since mechanical loading is a central feature of most clinical rehabilitation regimens, we next determined whether similar multiscale mechanisms in response to dynamic loading were present in healing tendon. Following injury, it is common for patients to complete physical therapy regimens that generally consist of joint movement^37^ and a progressive increase in mechanical loading over time. Using identical procedures as described above, we hypothesized that the multiscale mechanical, structural, and compositional properties of healing tissue would depend on the magnitude and duration of dynamic loading and the time since injury (Fig. 3a).

Similar to that seen for naïve tendons, loading magnitude affected tissue strain stiffening in healing tendon, as the change in equilibrium stress was reduced due to high magnitude, long duration loading compared to low magnitude loading (Fig. 3b). This decrease in strain stiffening was coupled with decreased dynamic modulus at 1% strain (Figure S8a,b) and elevated viscous dissipation (Figure S8c). Cycle number was a significant factor, regardless of applied load, on increasing tendon laxity (Figure S8d). Unlike uninjured tendons, however, the dynamic modulus in the healing tendon was affected by cycle number in high/1k groups (Figure S8e). Consistent with uninjured tendon, the response of healing tendon to dynamic loading showed elevated laxity during high/1k loading, which correlated with predicted fiber slack lengths (Figure S8f). Taken together, results indicate inferior fiber recruitment in healing tendons compared to uninjured tendons^38^.

### Dynamic Loading Differentially Affects Microscale Tendon Properties in Healing Tendon

To determine whether macroscale mechanical changes observed during dynamic loading in healing tendon propagated to the microscale, microstructural and nuclear evaluation was evaluated (Fig. 3c). Notably, high magnitude loading does not produce the same effects as tendon healing, likely due to differences in pericellular and extracellular matrix properties and in response to loading between healing states. Unlike uninjured tendons, dynamic loading did not affect collagen disorganization during healing (Fig. 3d). Although healing had an effect on F-actin (Figure S9b) and cellularity (Figure S9d), dynamic loading did not (Figure S9a). Dynamic loading resulted in decreased nCSD at 2-weeks post-injury, but not at 6-weeks post-injury (Figure S9c). Interestingly, although high magnitude loading decreased ΔnAR in uninjured tendons, the opposite was observed in healing tendons 2-weeks post-injury (Fig. 3e).

### Matrix Disorganization, Nuclear Disorganization, Cellularity, and F-Actin Predict Nuclear Deformation under Load

The micromechanics of the pericellular matrix and the forces that cells exert against this microenvironment can have important physiological consequences, driving division, migration, and differentiation processes. From a previous study, collagen disorganization, nuclear disorganization, and F-actin were associated with tendon healing^39^. Using experimental data derived above (Figure S10a), we first investigated whether nAR could be predicted from macroscale and microscale properties. We hypothesized that nAR and ΔnAR would be predicted most by strain stiffening, collagen organization, and cellularity. Initial screening using bivariate correlation revealed that the change in equilibrium stress, dynamic modulus, cellularity, F-actin staining, matrix disorganization, nuclear disorganization, and healing all correlated with the baseline nAR, with correlation coefficient magnitudes ranging from r = 0.33 to r = 0.85 (Table S1). Using these parameters, we conducted backward linear multiple regression. Results of the regression model found that cellularity, nuclear disorganization, and healing were significant predictors of nAR (R² = 0.85, p < 0.001; DW = 2.074) (Table S2).

Given this relationship to nAR, we next wanted to determine if the change in nAR, ΔnAR, could be predicted using these multiple regression models. ΔnAR is indicative of the degree to which nuclei deform in response to applied loading. Initial screening using bivariate correlation determined that ∆s_eq_, tanδ, cellularity, F-actin, ∆nCSD, healing, and high magnitude loading were significantly correlated to ΔnAR (Table S3). Surprisingly, predictions of ΔnAR were not increased greatly through inclusion of the structural properties in the multiple regression analysis, as the categorical variables healing and fatigue loading were the strongest predictors (R² = 0.39, p < 0.001; DW = 1.61) (Table S4). It is possible that there are competing effects due to the dynamic loading regimen. For example, fatigue loading reduces ΔnAR, along with ΔCSD and ΔnCSD^39^, unlike low magnitude dynamic loading.

### Tendon Cells in Uninjured Tissues Transmit and Sense Forces Further than in Healing and Fatigue Loaded Tissues: Model Predictions

**Figure 4:**
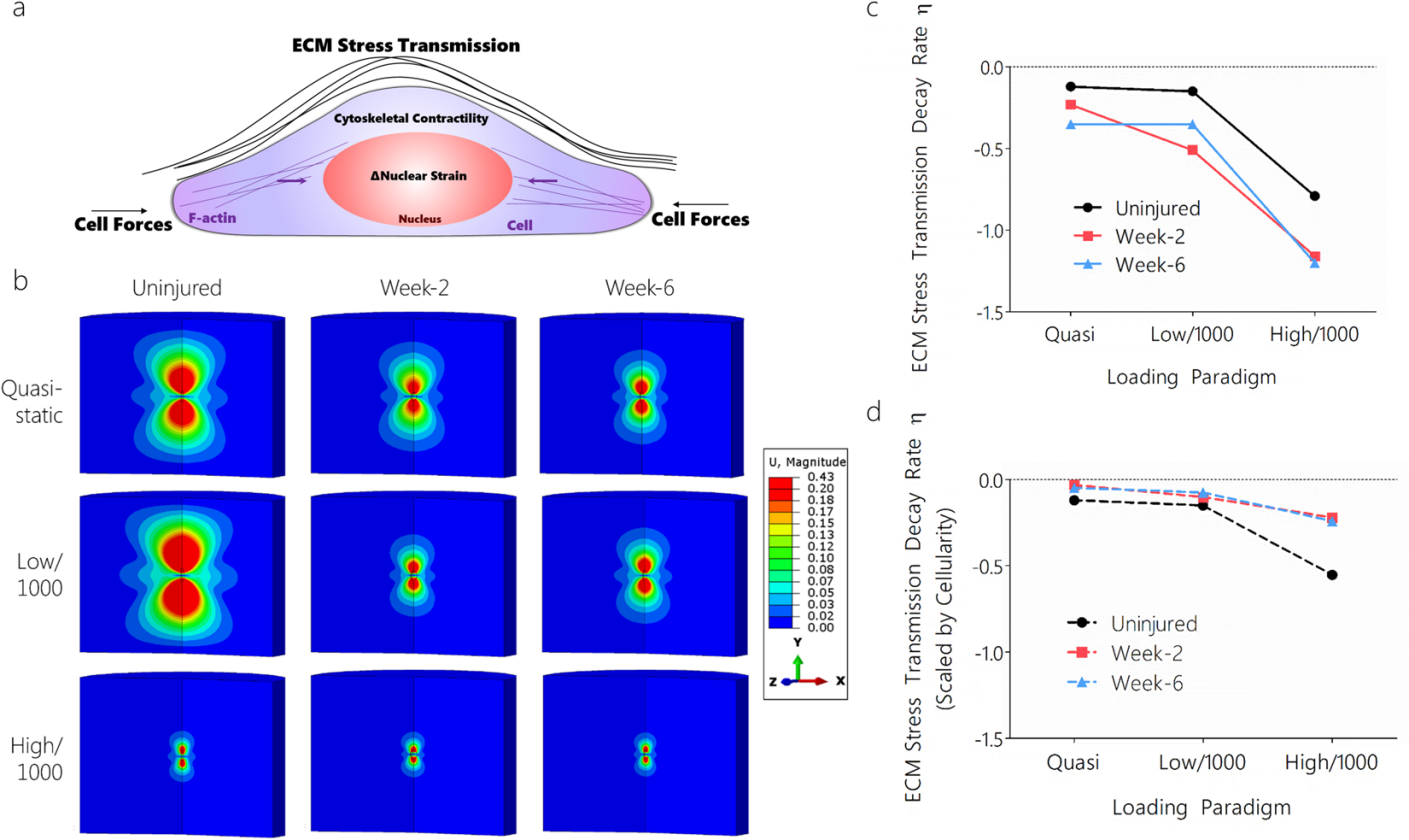
Tendon healing and dynamic loading affect cell-generated stress transmission through the surrounding ECM. (**a**) Cell-generated stress transmission through the surrounding ECM decayed more rapidly in (**b**) healing tendon and in tendons subjected to high magnitude dynamic loading, as evidenced by (**c**) the decreased exponent of displacement decay *η*. (**d**) When scaling the ECM stress transmission decay rate by tissue cellularity, the effective ECM stress transmission decay rate was more similar between groups. U indicates displacment (μm) from the cell perimeter. “Quasi” indicates quasi-static loading, “low/1k” indicates low magnitude loading for 1000 cycles, and “high/1k” indicates high magnitude loading for 1000 cycles.

Cells actively probe their microenvironment via contractile forces generated and exerted against their immediate tissue environment. The surrounding tissue properties define how far into the ECM cells can transmit and receive mechanical forces. Therefore, we investigated how healing and dynamic loading regulate cell engagement and ECM stress transmission (i.e., distance by which cells can feel substrate stiffness or other cells)^21^ through the ECM (Figure S10b). Cell-generated stress transmission to the ECM may have important ramifications for both cell-cell communication and load transfer and influence continued maintenance or failure to heal following injury by altering cellular perception of the microenvironment. We hypothesized that both healing and high magnitude dynamic loading would decrease cell-generated stress transmission through the surrounding ECM. We first investigated how displacements were transmitted in healing tendon compared to uninjured intact tendon. Model input parameters were determined by fitting stress-stretch relationships to experimental data (Figure S11). Using these inputs, we applied varying levels of cell contraction in an axi-symmetric model. For the uninjured quasi-static loading case, the model mesh converged and remaining simulations proceeded with mesh densities of 1 µm local to the cell that increased to 6 µm at 100 µm from the cell. Model parameters were input for the test conditions and two independent models were generated encompassing the appropriate tendon cell geometry and material properties. In both cases, the cell was allowed to contract 5% (95% of original volume) and the profile of displacement transmission (μm) was quantified as a function of distance from the cell, *r* (Figure S12). The u magnitude displacement indicated in subsequent figures is therefore relative to the cell perimeter of the contracting cell. Cell aspect ratios were incorporated into our modeling framework as they have been shown to contribute towards ECM stress transmission (contraction of spindle cells transmits stress more than rounded cells)^21^. We found that, in healing tendon, displacement transmission profiles decreased substantially compared to uninjured intact tendon (Fig. 4a). As the tendon cell contracted, stresses were transmitted distances almost 10x the cell radius. However, in healing tendon, these profiles decayed much more quickly. The displacement profiles followed a power law decay^21^, *u(r)*∝*r*^−*η*^.

We next investigated how displacements are transmitted in fatigue loaded tendon compared to uninjured intact tendon (Fig. 4a). As done previously, model parameters were input and tendon contraction (5%) was simulated. Strikingly, fatigue loaded tendons demonstrated drastic decreases in simulated ECM stress transmission compared to naïve tendons (Fig. 4b). Regardless of the applied cell contraction, fatigue loaded tendon displacement profiles decayed rapidly within short distances from the cell surface (Fig. 4c). These large differences are likely attributed to an elongated toe-region that results from the repeated high magnitude loading. Although fatigue loading promoted multiscale strain transfer in early healing, modeling did not predict similar improvements in ECM stress transmission. The high cellularity present in healing tendons may be a mechanism to re-establish multiscale strain transfer in early healing and cell-cell communication. Indeed, when scaling the ECM stress transmission decay rate by tendon cellularity, the effective ECM stress transmission decay rate was more similar between groups (Fig. 4d). Taken together, both tendon healing and fatigue loading attenuate cell generated stress transmission through the ECM compared to uninjured tendon.

## Conclusions

**Figure 5:**
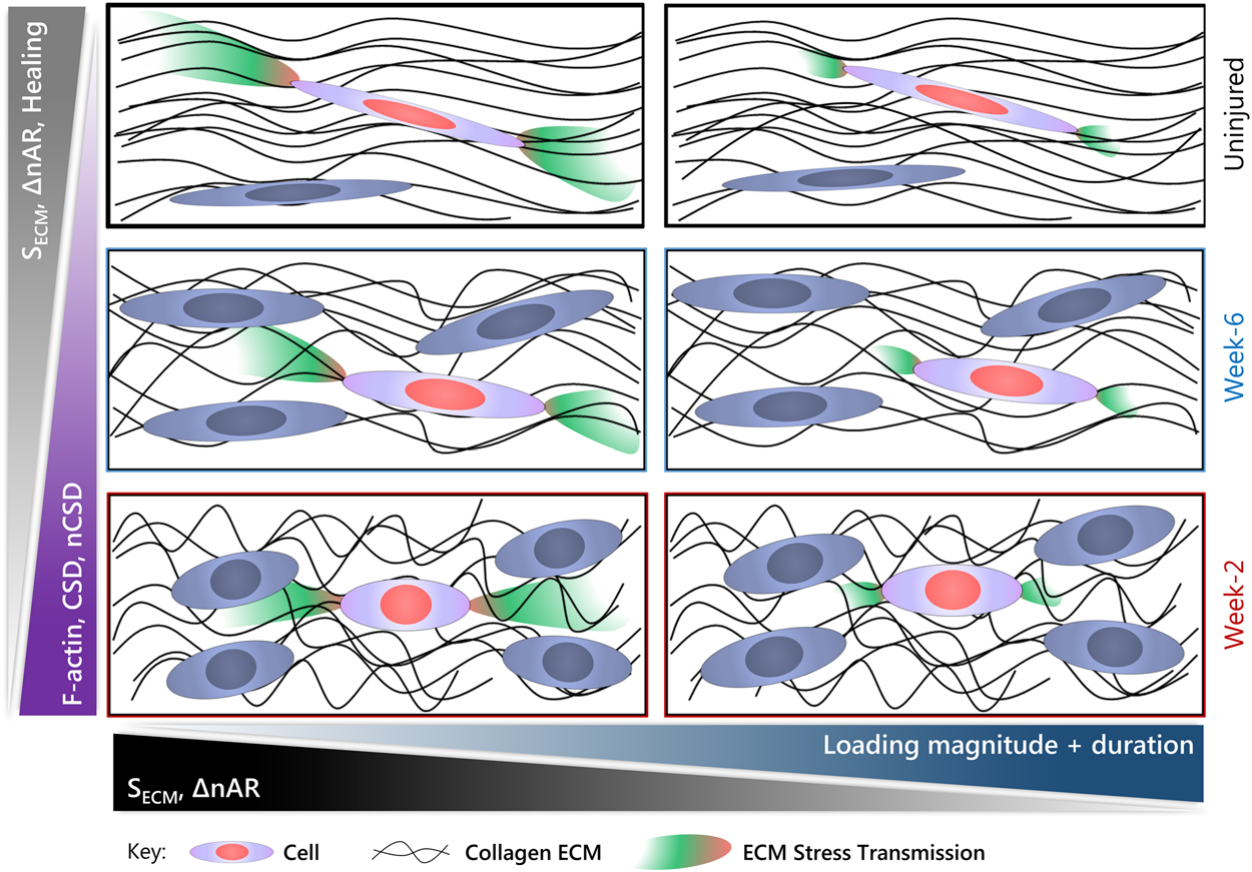
Dynamic loading and tendon healing affect multiscale tendon properties and cell-generated stress transmission through the ECM. Summary of findings highlighting differences in matrix stress transmission (S_ECM_, color gradients), change in nuclear aspect ratio with applied strain (ΔnAR), healing, F-actin, matrix disorganization (CSD), nuclear disorganization (nCSD), loading magnitude, and loading duration. Each row indicates a healing group. The left column is the response during quasi-static loading, in contrast to the right column during high magnitude long duration loading.

This study evaluated multiscale structure-function mechanisms in living tendon explants in response to dynamic tensile loading (varying magnitude and duration). After establishing multiscale relationships in uninjured tendon, we evaluated whether similar features would exist in healing tendon, using an established patellar tendon injury model^39–41^ and whether these multiscale mechanical, structural, and compositional properties could predict the cell-level deformations (change in nAR with applied strain) using multiple regression analysis. Together, this work identified specific cell and ECM properties that affect the tendon multiscale response to dynamic loading and during healing, and predicted how dynamic loading and healing subsequently affect the ability of tendon cells to query the local and distant properties of the tendon through cellular engagement with the ECM (Fig. 5).

We first showed that tendon strain stiffening is reduced, and non-recoverable, due to high magnitude long duration loading and occurs in concert with increased laxity and delayed fiber re-alignment with applied strain. These macroscale properties correlated to microscale properties; high magnitude long duration loading resulted in increased collagen and nuclear disorganization at high strains, and decreased change in nAR with applied loading. Baseline nAR was strongly correlated with many macroscale and microscale properties, highlighting its relationship to multiscale tendon properties. nAR had the highest correlations to CSD, F-actin staining, and healing. Both tendon healing and fatigue loading affected the transmission of cell-generated stress through the surrounding matrix. Overall, results indicate that a major hurdle to regain native multiscale phenotypes in tendon is to restore strain transfer to increase the change in nuclear deformation during applied loading and to regenerate a matrix capable of long range ECM stress transmission. It is possible that tendon cells restore ECM stress transmission, in part, by increasing in cell number initially in early healing and the amount of F-actin per cell^39^. Taken together, we highlight numerous mechanical, structural, and compositional properties that contribute to the dynamic capacity of tendon cells to respond to loading and transmit stresses and probe ECM properties over long distances.

Although chondrocyte nuclei have been shown to be shape and volume sensitive to compression induced changes since 1995^42^, tenocyte nuclei were first observed to be strain responsive in rat tail in 2002^43^. Nuclear shape and organization are important because mechanical forces applied to and deformation of the nucleus can regulate transcriptional activity^3,44^ and protein synthesis^3^. Relations between the ECM and structural organization of the nucleus can further modulate tissue phenotype^4^. This study did not investigate proteins that may promote or impede strain transfer (e.g., ECM, cell-ECM, pericellular, and cell proteins). During loading, strain transfer to the nucleus can direct access of chromatin to transcriptional regulators, as nascent RNA synthesis is detected in interchromatin regions^14^. Another mediator of nuclear deformation are A-type lamins, which affect nucleus structure, shape and stability^45,46^. Nuclear strain transfer may also affect Yes-Associated-Protein (YAP) signaling (a mechanosensing pathway that relays mechanical input via translocation to the nucleus)^47^.

This work is not without limitations. First, we only investigated tensile loading, and adding in additional loading paradigms that apply shear, compression, and biaxial forces may provide further insight into behavior *in vivo*. Next, we investigated the multiscale response at two distinct strains (1% and 10%). These strains were chosen to represent the toe and linear portion of the force-displacement curve. However, it is well established that the tendon response to loading is inherently nonlinear; therefore, evaluation at additional strains would help to fully describe the responses presented. Due to the mode of injury, maintenance of tendon struts may result in stress shielding in adjacent healing tissues. However, these struts are necessary to prevent extensive tendon lengthening and may provide similar protection to tendon repair approaches. Our multiscale evaluation focused solely on the healing injury site through use of tissue stamping^48^, but it is also possible that the pseudo-normal tissue flanking the injury window may also be interesting to examine in future studies. Although we employed many tools to assess multiscale tendon properties, other measurements may provide additional support for our findings. It is likely that other metrics of micro/nano damage may be occurring in our cyclic loading model. Indeed, previous studies in this space have evaluated surrogate measures of tendon damage including fibril denaturation^49^, crimping^50^, fibril kinking^9^, and sliding^51^. Additionally, it is possible that other cell microenvironmental changes in the ECM and pericelluar matrix may be present during healing and cyclic loading that alter the multiscale response to loading including water content, pH, redox, hypoxia, ATP, glucose, enzymes, and temperature. Low and moderate loading have been shown to have no effect on water content^27^ or GAG production^32^, whereas longer periods of loading do influence GAG content^30^, and overloading may cause lower strength and release of proinflammatory cytokines such as PGE_2_ and NO^26^. An optimal loading regime has been proposed to promote mechanics^29^, potentially through collagen synthesis^16,32^ as the molecular mechanisms switch from a catabolic to anabolic response^33^. Such mechanisms may be frequency and duration dependent^28^.

This work supports a new understanding for the implications of macroscale mechanical loading on multiscale tendon properties in uninjured and healing tendon. Ultimately, this work furthers our understanding of the tendon multiscale response to loading, provides a framework for the micromechanical environment that tenocytes interact with in response to dynamic loading and healing, and establishes important benchmarks for tendon tissue engineering. The multiscale response to mechanical loading, which is a central feature of clinical rehabilitation protocols, is necessary to determine the ramifications of various macroscale loading regimens. Additionally, these results provide information as to the nature of the environment that therapeutic cells may experience following cell delivery therapies. Several exciting future avenues of research are possible that would highly impact basic science research of tendon function and lead to translatable approaches that could improve tendon injury onset and healing response.

## Methods

### Surgical Model

All methods and procedures were carried out in accordance with guidelines and regulations by the University of Pennsylvania Institutional Animal Care and Use Committee. Female C57BL/6 mice (N = 188) at 150 days of age were randomized into an uninjured control group and groups that received bilateral injury to their patellar tendons (N = 375 total tendons)^40,52,53^. Mice in injury groups were first anesthetized (isoflurane) and both hindlimbs were shaven and sterilized. Using aseptic procedures, a single incision was made through the skin near the knee, and longitudinal incisions were made adjacent to and on either side of the patellar tendon. A rubber-coated backing was placed under the tendon, and a full thickness, partial width (~60% width) excisional injury was created using a 0.75 mm biopsy punch. Skin was closed with 5-0 prolene suture, and the animals were returned to cage activity. Doses of buprenorphine (0.1 mg/kg; Penn Investigational Drug Service) were given pre-surgery, and sustained-release buprenorphine (1.0 mg/kg; ZooPharm; Windsor, CO) was given post-surgery. Injured animals were randomized into groups euthanized at 2 or 6 weeks post-injury to evaluate the role of healing on multiscale tendon properties. At these time points, mice were sacrificed and tendons were mechanically tested *ex vivo* with tissue tendon explants.

### Sample Preparation and Mechanical Testing

For multiscale evaluation relating applied macroscale mechanical loading to microscale properties (cell, nuclear, and collagen morphology), tendons were harvested immediately after sacrifice and kept in standard media conditions to maintain cell viability (Figure S2a). Maintenance of cell viability was important to preserve *in vivo* cell contractile activity during loading, which are ATP driven processes^54^. The patellar tendon was stamped into a “dog-bone” shape to isolate the injury site, and measured for cross sectional area at the injury site^55^. To maintain tenocyte viability during loading, tissues were immersed in a bath containing sterile DMEM supplemented with 5% fetal bovine serum (FBS), maintained at 37 °C integrated with a tensile testing device (Instron 5848; Norwood, MA). To evaluate the effect of dynamic loading and healing on strain stiffening, tendons were randomized into a zero, low, or high magnitude loading protocol (corresponding to the toe or linear regions of the force-displacement curve) for either 10 or 1000 cycles at 1 Hz (Groups: Low/10, Low/1k, High/10, High/1k) (Figure S2b). Loading levels were determined from ramp-to-failure data derived from uninjured and healing patellar tendons, with loading regimes representing the transition (low) and linear (high) portions of the load-displacement curve. Tendons (n = 10–13/group) were preconditioned and ramped at constant strain rate (0.1% strain/s) until 1% or 10% strain prior to a frequency sweep (0.125% strain amplitude at 0.1, 1, 5, and 10 Hz) and snap freezing.

During loading, force and displacement data were acquired from 100–1000 Hz and analyzed using custom MATLAB code (Mathworks, Natick, MA). Several post processing parameters are computed both during the mechanical and diagnostic tests: 1) maximum/minimum cyclic displacement and strain; 2) tangent stiffness (calculated as the slope between the maximum and minimum force and displacements for each cycle); 3) stress (calculated as the force divided by the cross sectional area); 4) dynamic modulus (calculated as the slope between the maximum and minimum stress and strain for each cycle; 5) hysteresis (defined as the area enclosed by the stress-strain curve for a given cycle); and 6) laxity (defined as the ratio of displacement and gauge length relative to the first cycle of fatigue loading, and assessed at constant load throughout fatigue testing). Because laxity was defined relative to the initial cycles of loading, comparisons between loading groups are not possible and are therefore made within loading magnitudes (low or high). In addition to properties quantified during dynamic loading, we evaluated dynamic properties at 1% and 10% strain. The change in equilibrium stress (force divided by the cross sectional area) between 1 and 10% strain was used to indicate the amount of strain stiffening, and the dynamic modulus and phase shift assessed during frequency sweeps were computed.

For evaluation of macroscale tendon structure, a separate cohort of tendons (N = 10/group) were frozen at −20 °C, thawed, and fine dissected as done previously^40,41^. Patella–patellar tendon–tibia complexes were carefully isolated by dissection under magnification and tendons were carefully stamped into a “dog-bone” shape of width 0.75 mm. Before and after stamping, tendon cross-sectional area was measured with a custom, laser-based device^55^. The distal half of the tibia was then secured in a custom fixture and loaded into a material testing system (Model 5848, Instron; Norwood, MA) with a 10 N load cell. During loading, force and displacement data were acquired and analyzed using MATLAB (Mathworks, Natick, MA). In addition, these experiments were used to evaluate the role of recovery. Following cyclic loading, tendons were maintained at 0% strain for 1000 s^56^. This recovery period is commonly used in tendon, and ranges from 1 – 45 minutes^56–60^. Following recovery periods, tendons were subjected to the same quasi-static loading protocol to both 1 and 10% strain during which polarized light images were taken.

### Assessment of Cell Viability

Cell viability was evaluated with an MTT assay, which exploits the redox potential of actively respiring cells (Figure S3). In live cells, water-soluble MTT is converted to an insoluble purple formazan, which is solubilized and determined for concentration by optical density. A 12 mM MTT stock solution was prepared by adding 1 mL of sterile PBS to 5 mg of MTT under sterile conditions. For labeling viable cells in tissue, 100 µL of stock solution is added to 1000 µL of DMEM, keeping the mixture at 37 C at 5% CO_2_ prior. Following tissue harvest, tissue was placed immediately in Eppendorf tubes left open in an incubator for 1-hour. Tissues were transferred to 1xPBS for 30 min at room temperature and fixed with 4% PFA at 4 °C overnight prior to cryosectioning. Tissue sections were counter stained with DAPI to qualitatively compare viable cells with all cells.

### Polarized Light Imaging

A polarized light system^7^ was integrated with the mechanical testing, consisting of a backlight, 90° offset rotating polarizer sheets (Edmund Optics, Barrington, NJ) on both sides of the test sample (polarizer and analyzer), and a GigE aca2040gm camera (resolution: 2048 × 2048 pixels) (Basler, Exton, PA). Sets of alignment maps (30 images) were taken during the quasi-static ramp and following frequency sweeps at 1% or 10% strain. With these data, we assessed the strain at which collagen fiber re-alignment occurred by determining the transition point of a bilinear fit using CSD versus strain data^61^.

A custom MATLAB program (MATLAB, Natick, MA) divided images into a series of regions of 100 pixels² spaced at 20 pixels and individually averaged to filter noise. From these data, the signal phase and magnitude, within each region from each alignment image series, was used to determine the circular standard deviation (CSD), a measure of collagen fiber disorganization^62^. Briefly, the CSD was calculated by fitting a sin²(2θ) function^63^ to the pixel intensity-polarizer angle data to determine the angle corresponding to the minimum pixel intensity. This angle represents the average direction of fiber alignment.

### Fiber Recruitment Model

We applied a structurally based elastic model^64–66^ to our mechanical data to quantify the non-linear force-displacement behavior as fibers uncrimp to their slack length. This model assumes that fiber uncrimping gives rise to tendon’s non-linear force-displacement behavior, and is modeled using a cumulative probability distribution function (Eq. 1). Briefly, *p* is the cumulative probability of a fiber being uncrimped (range: 0–100%), *σ* is the standard deviation of fiber slack-lengths (mm), *L*_0_ is the fiber slack length (mm), and *μ* is the average fiber slack length (mm). Fibers are considered uncrimped once the tendon length has exceeded the fiber’s slack-length (Eq. 2). Here, *K*_avg_ is the average fiber stiffness (N/mm), x is the tendon length (mm), *L*_0_^*i*^ is the slack length of fiber i, and *H* is the Heaviside step function (*H* = 0 for *x* < *L*_0_^*i*^ and 1 for *x* > *L*_0_^*i*^). Disorganized tissues, such as skin, have an elongated toe-region, which results in slack lengths with high means and standard deviations.

1 $$p({L}_{0})=\frac{1}{\sigma \sqrt{2\pi }}{\int }_{-\infty }^{{L}_{0}}{e}^{\frac{-{(t-\mu )}^{2}}{\sigma }}$$

2 $$F(x)={K}_{{\rm{avg}}}\sum _{i=1}^{N}(x-{L}_{0}^{i})\cdot H(x-{L}_{0}^{i})$$

### Immunofluorescent Staining

After each loading protocol, tissues were snap frozen, and immediately embedded in Optimum Cutting Temperature (OCT) compound (Tissue-Tek, Sakura Finetek USA, Torrance, CA, USA), prior to sectioning at 15μm (Leica CM1950; Wetzlar Germany) using Kawamoto’s Film^67^. Following cryosectioning, sections were placed in 4% paraformaldehyde (Fisher) for 3 minutes and attached to slides with a chitosan film adhesive solution. Tissue sections were rehydrated in 1xPBS, blocked for non-specific binding in 5% BSA and 0.1% triton for 1-hour at 4 °C, and then stained with primary conjugated antibodies for cell and nuclear shape analysis. Sections were stained for F-actin with Alexa Fluor® phalloidin (1:20; 555/565; Fisher Scientific), and nuclei with DRAQ5 Fluorescent Probe Solution (1:1000; 647/681; Fisher Scientific) for 12 hours at 4 °C. Cell and nucleus long and short axes were measured using a custom MATLAB program and ImageJ (v1.48, NIH, Bethesda, MD) to derive aspect ratios.

### Confocal and Multiphoton Imaging

Sequential scans on an upright laser-scanning multiphoton confocal microscope (Leica TCS SP8; Wetzlar Germany; 1024 × 1024 pixel resolution, fov: 277 µm × 277 µm, scan speed: 400 Hz) were completed to evaluate stains for collagen, F-actin, and nuclei in tendon sections, as described previously^39^. Z-stacks were taken at 1.5 µm intervals to capture the tendon midsubstance or injury site. Images from 2–3 tendon sections were taken per specimen.

We determined the average collagen fiber organization by computing the angular orientation of fibers in the SHG images with custom code (MATLAB; v2015a; Natick, MA), as previously described^39,68^. Briefly, images were selected from the middle of each Z-stack with fibers aligned along the horizontal direction, images were contrast enhanced, and fiber alignment determined by Fourier analysis. A quiver plot was superimposed on the original image to ensure that collagen fiber orientation was computed correctly. Angles were plotted in a histogram to determine angular distribution. For average organization, the circular standard deviation was calculated at 1% or 10% strain to evaluate changes in fiber orientation during tendon loading. The average circular statistics was taken across all selected images in the Z-stack for uninjured samples.

### F-Actin and Nuclear Shape Analysis

F-actin was quantified by determining the percent positive staining per ROI. Images were imported into FIJI, cropped, max projected, converted to 8-bit greyscale, contrast enhanced, and thresholded to isolate F-actin filaments. The number of F-actin positive pixels was determined and normalized to the total cropped ROI. The same ROIs for nuclei were similarly selected in FIJI, and segmented and analyzed using CellProfiler^36^ to evaluate nuclear shape (major/minor axis, nuclear aspect ratio (nAR)) and disorganization (nCSD).

### Statistical Analysis

Data normality was assessed and confirmed with Shapiro Wilk tests. To determine the role of loading magnitude and duration on multiscale tendon properties, data were evaluated with two-way ANOVAs with post hoc Student’s t-tests with Bonferroni corrections (SPSS, Armonk, NY USA). For comparisons between healing time points, the reader is referred to our recent manuscript^39^. To determine the role of strain on tendon multiscale properties, data were evaluated with Student’s t-tests with Bonferroni corrections. Linear regression was used to determine relationships between microscale structure, composition, and nuclear shape.

### Multiple Regression Analysis

Prior to model incorporation, data were examined for outliers, defined as being at least 2.2*IQR above (below) the third (first) quartile^69^. The *R*^2^ was chosen assuming the current study would predict mechanical parameters better than previous studies^70,71^. Multiple regression analysis assumed that each dependent and independent parameter was obtained from a single specimen (a mouse).

Summary statistics of all variables were examined to ensure that assumptions for linear analysis were satisfied. Pearson’s correlations were calculated between independent variables. A general linear model (GLM) was evaluated to determine if dependent variables (nAR and ΔnAR) were significantly related to the independent parameters: equilibrium stress (s_eq_), the change in s_eq_ from 1–10% strain, |E*|, tanδ, collagen disorganization (CSD), the change CSD from 1–10% strain (ΔCSD), cell number, F-actin, fiber mean slack length (SL_mn), nuclear disorganization (nCSD), and the change in nCSD from 1–10% strain (ΔnCSD). These properties were measured after two cyclic loading protocols (low magnitude and high magnitude) at two strains (1 and 10%) for three treatment groups (uninjured, healing 2-weeks post-injury, healing 6-weeks post-injury). Data from both strain levels were pooled for subsequent analysis to identify key variables and create model fits used to predict responses in a subset of the data. This same process was repeated for each treatment and mechanical loading group.

Backward linear regression was performed (probability of F to enter is set at 0.05 and to be removed is set at 0.10)^72,73^. The criteria for choosing the appropriate range of F to enter and F to remove were based on the degrees of freedom in the model. This range for F corresponds to a significance level of 0.1 for a single test. The tolerance level, defined as 1-R_k_², was set to 0.01 to prevent entry of variables that are highly correlated to other x-variables. In other words, a variable was excluded if its coefficient of multiple determinations, when regressed over the other x-variables, exceeds 0.99. Significance was again set at p < 0.05, per regression model and variances within the data. The Durbin Watson (DW) statistic was calculated to identify the existence of correlations between independent variables. For each equation, based on established guidelines for interpretation of DW statistic, a DW value lower than 1.08 indicates correlation between the independent variables^72,73^.

### Modeling ECM Stress Transmission

We applied a constitutive law for fibrous matrices^21^ to model stress transmission in tendon using experimental inputs. Finite-element simulations of this constitutive law were used to study the effect of material properties of the isotropic (*E*_b_) and fibrous (*E*_f_) components of the matrix, the shape of cells, and the polarization of cell contractile forces on force transmission in fibrous matrices in response to dynamic loading and healing. In this constitutive law, two distinct groups of aligned and isotropic fibers were incorporated. Isotropic fibers were modeled as neo-Hookean hyperelastic^21^, and energy functions describing aligned fibers were chosen so that the tendon matrix increases in stiffness under tensile loading^21^. Briefly, the Cauchy stress was decomposed into isotropic (**σ**^b^) and fibrous (**σ**^f^) contributions (Eqs 3–6)^21^. The amount of interaction between the two families of fibers is given by the ratio, of *E*_f_/*E*_b_^21^. Previous work showed that this model captured the essential features of discrete fiber simulations during mechanical loading, such as the toe in the stress strain curve^21,74^.

3 $${\boldsymbol{\sigma }}={{\boldsymbol{\sigma }}}^{{\bf{b}}}+{{\boldsymbol{\sigma }}}^{{\bf{f}}}$$

4 $${{\boldsymbol{\sigma }}}^{{\bf{b}}}=\kappa (J-1){\bf{I}}+G\mathrm{dev}(\bar{{\bf{B}}})/J$$

5 $${{\boldsymbol{\sigma }}}^{{\bf{f}}}=\frac{1}{J}\sum _{a=1}^{3}\frac{\partial f({\lambda }_{a})}{\partial {\lambda }_{a}}{\lambda }_{a}({{\boldsymbol{n}}}_{{\boldsymbol{a}}}\otimes {{\boldsymbol{n}}}_{{\boldsymbol{a}}})$$

6 $$\frac{\partial f({\lambda }_{a})}{\partial {\lambda }_{a}}=\{\begin{array}{cc}0 & {\lambda }_{a} < {\lambda }_{1}\\ \frac{{E}_{{\rm{f}}}{(\frac{{\lambda }_{a}-{\lambda }_{1}}{{\lambda }_{2}-{\lambda }_{1}})}^{n}({\lambda }_{a}-{\lambda }_{1})}{n+1}, & {\lambda }_{1}\le {\lambda }_{a} < {\lambda }_{2}\\ {E}_{{\rm{f}}}[\frac{{\lambda }_{2}-{\lambda }_{1}}{n+1}+\frac{{(1+{\lambda }_{a}-{\lambda }_{2})}^{m+1}-1}{m+1}], & {\lambda }_{a}\ge {\lambda }_{2}\end{array}$$

For finite element implementation of this constitutive law (Eqs 1–4), several parameters were included: *λ*_*a*_ are the principal stretches, *λ*_1_ and *λ*_2_ are the critical stretches, which demarcate the different regimes of the stress-strain curve and *m* and *n* are the exponents used to model the matrix strain-stiffening. *κ* is the initial bulk modulus, and *G* is the initial shear modulus, ***n***_*a*_ are the unit vectors in the principal stretch orientations, **C** (**C = F**^**T**^**F**) and **B** (**B = FF**^**T**^) are the right and left Cauchy-Green deformation tensors where **F** is the deformation gradient tensor, and *J* is its Jacobian (*J* = det(**F**))^21^. Specific tendon cell aspect ratios and strain polarizations, were input from experimental data. Simulations were completed using Abaqus/CAE (Dassault Systems, 2016) in a finite deformation setting, with matrices modeled using 3-node bilinear axisymmetric tri elements^21^. The material form of the tangent modulus tensor **C**^**SC**^, the tangent modulus tensor for the convected rate of the Kirchhoff stress **C**^**τC**^, the tangent modulus tensor for the Jaumann rate of the Kirchhoff stress **C**^**τJ**^, and the material Jacobian **C**^**MJ**^ for the material model were implemented^21^. The principal stretches are evaluated as the square roots of the eigenvalues of **C**.

## Electronic supplementary material

Supplemental Information
