# Supplementary material for: Dynamic Loading and Tendon Healing Affect Multiscale Tendon Properties and ECM Stress Transmission
Source: Sci Rep. 2018 Jul 18;8:10854. doi: 10.1038/s41598-018-29060-y (PMC6052000; doi:10.1038/s41598-018-29060-y)
Supplement: Supplementary file 1 — Supplemental Information [file 41598_2018_29060_MOESM1_ESM.docx]

**Dynamic loading and tendon healing affect multiscale tendon properties
and ECM stress transmission**

Running title: Multiscale response of tendon to loading during healing and dynamic loading

^1,2,5,6^Benjamin R Freedman, ^1^Ashley B Rodriguez, ^1,2^Ryan J Leiphart, ^1,2^Joseph B Newton, ^3^Ehsan Ban, ^4^Joseph J Sarver, ^1,2^Robert L Mauck, ^3^Vivek B Shenoy, ^1,2^Louis J Soslowsky

^1^McKay Orthopedic Research Laboratory, University of Pennsylvania, Philadelphia, PA

^2^Department of Bioengineering, University of Pennsylvania, Philadelphia, PA

^3^Department of Materials Science, University of Pennsylvania, Philadelphia, PA

^4^Department of Biomedical Engineering, Drexel University, Philadelphia, PA

^5^John A. Paulson School of Engineering and Applied Sciences, Harvard University, Cambridge, MA

^6^Wyss Institute for Biologically Inspired Engineering, Harvard University, Boston, MA

**Supplemental Information**

**Table S1: Correlations to nAR.** Δs_eq_, |E*|, cellularity, F-Actin, CSD, nCSD, ΔnCSD, and healing were significantly correlated to nAR (p<0.0001). Parameters without p-values were non-significant.

|  |  | **∆s_eq_** | **\|E*\|** | **tanδ** | **Cellularity** | **F-Actin** | **CSD** | **∆CSD** | **nCSD** | **∆nCSD** | **Heal** | **High Mag** |
| --- | --- | --- | --- | --- | --- | --- | --- | --- | --- | --- | --- | --- |
| **nAR** | **Pearson**  **Correlation** | 0.40 | 0.33 |  | -0.74 | -0.83 | -0.85 |  | -0.81 |  | -0.89 |  |
|  | **Sig. (2-tailed)** | <0.0001 | <0.0001 |  | <0.0001 | <0.0001 | <0.0001 |  | <0.0001 |  | <0.0001 |  |
|  | **N** | 91 | 172 |  | 176 | 170 | 178 |  | 175 |  | 181 |  |

**Table S2: Multiple regression modeling to predict nAR.** Cellularity, nCSD, and healing were the strongest predictors of nAR (R^2^ = 0.85).

| **Parameter** | **β** | **t** | **sig** |
| --- | --- | --- | --- |
| **Constant** | -0.40 | -0.12 | 0.903 |
| **Cellularity** | 1.81 | 2.61 | 0.011 |
| **nCSD** | -2.44 | -4.40 | <0.0001 |
| **Healing** | -4.22 | -8.60 | <0.0001 |

**Table S3: Correlations to ΔnAR.** Δs_eq_, tanδ, cellularity, F-Actin, ΔnCSD, healing, and high magnitude loading were significantly correlated to ΔnAR (p<0.003). Parameters without p-values were non-significant.

|  |  | **∆s_eq_** | **\|E*\|** | **tanδ** | **Cellularity** | **F-Actin** | **CSD** | **∆CSD** | **nCSD** | **∆nCSD** | **Heal** | **High**  **Mag** |
| --- | --- | --- | --- | --- | --- | --- | --- | --- | --- | --- | --- | --- |
| **ΔnAR** | **Pearson**  **Correlation** | 0.34 |  | -0.33 | -0.43 | -0.30 |  |  |  | -0.32 | -0.39 | -0.36 |
|  | **Sig. (2-tailed)** | <0.001 |  | <0.001 | <0.001 | 0.003 |  |  |  | <0.001 | <0.001 | <0.001 |
|  | **N** | 89 |  | 96 | 98 | 94 |  |  |  | 98 | 100 | 100 |

**Table S4: Multiple regression modeling to predict ΔnAR.** Healing and high magnitude loading (fatigue) were the strongest predictors of ΔnAR (R^2^ = 0.39).

| **Parameter** | **β** | **t** | **sig** |
| --- | --- | --- | --- |
| **Constant** | 1.50 | 9.94 | <0.0001 |
| **Healing** | -1.05 | -5.86 | <0.0001 |
| **Fatigue** | -0.63 | -3.36 | 0.001 |

**
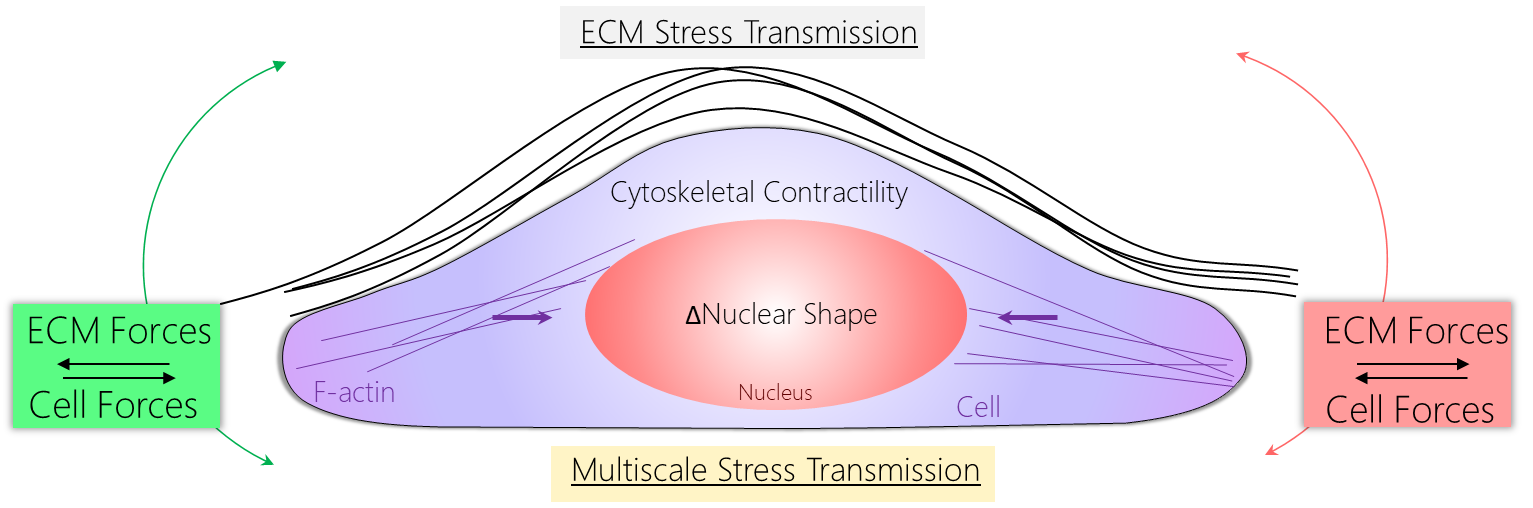
**

**Figure S1: ECM and cell forces affect multiscale stress transfer and ECM stress transmission.** Conceptual overview highlighting the dynamic reciprocity between ECM and cell forces driving multiscale stress transmission and ECM stress transmission. Imbalance in ECM and cell forces created during tendon injury and as a consequence of dynamic loading may feedback to promote healthy or pathologic tendon cell and tissue phenotypes.

**
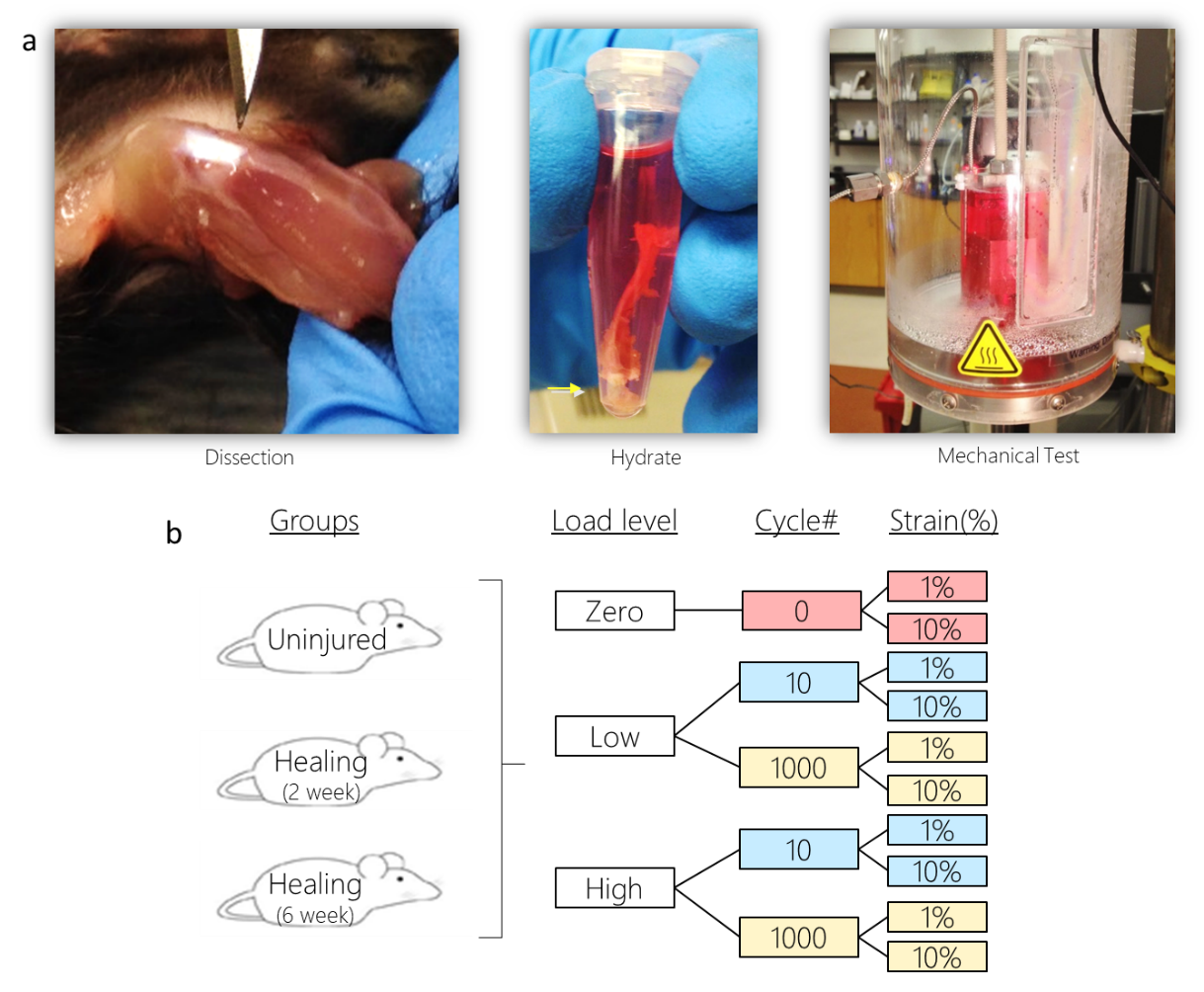
**

**Figure S2: Experimental setup for live tendon testing.** (**A**) Patellar tendons were immediately harvested, stamped into a dog bone shape, and maintained hydrated in basal media prior to mechanical loading. (**B**) The three groups (uninjured, 2-week post-injury, and 6-weeks post-injury) underwent several mechanical testing protocols that varied both the magnitude (load level) and duration (cycle #) of loading prior to snap freezing at 1 or 10% strain and subsequent property multiscale evaluation.

**
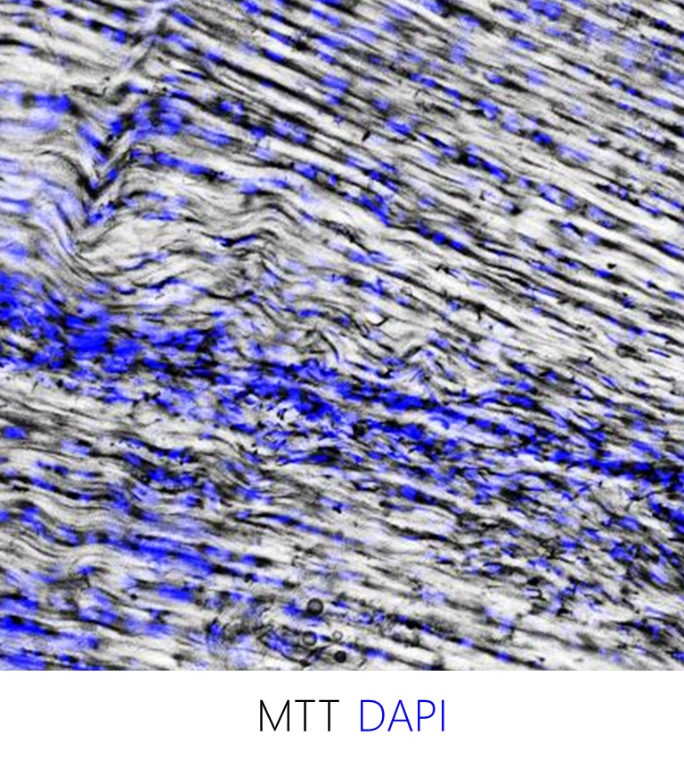
**

**Figure S3:** **Cell viability.** Cells remained viable (MTT: black; DAPI: blue) following mechanical loading.

**
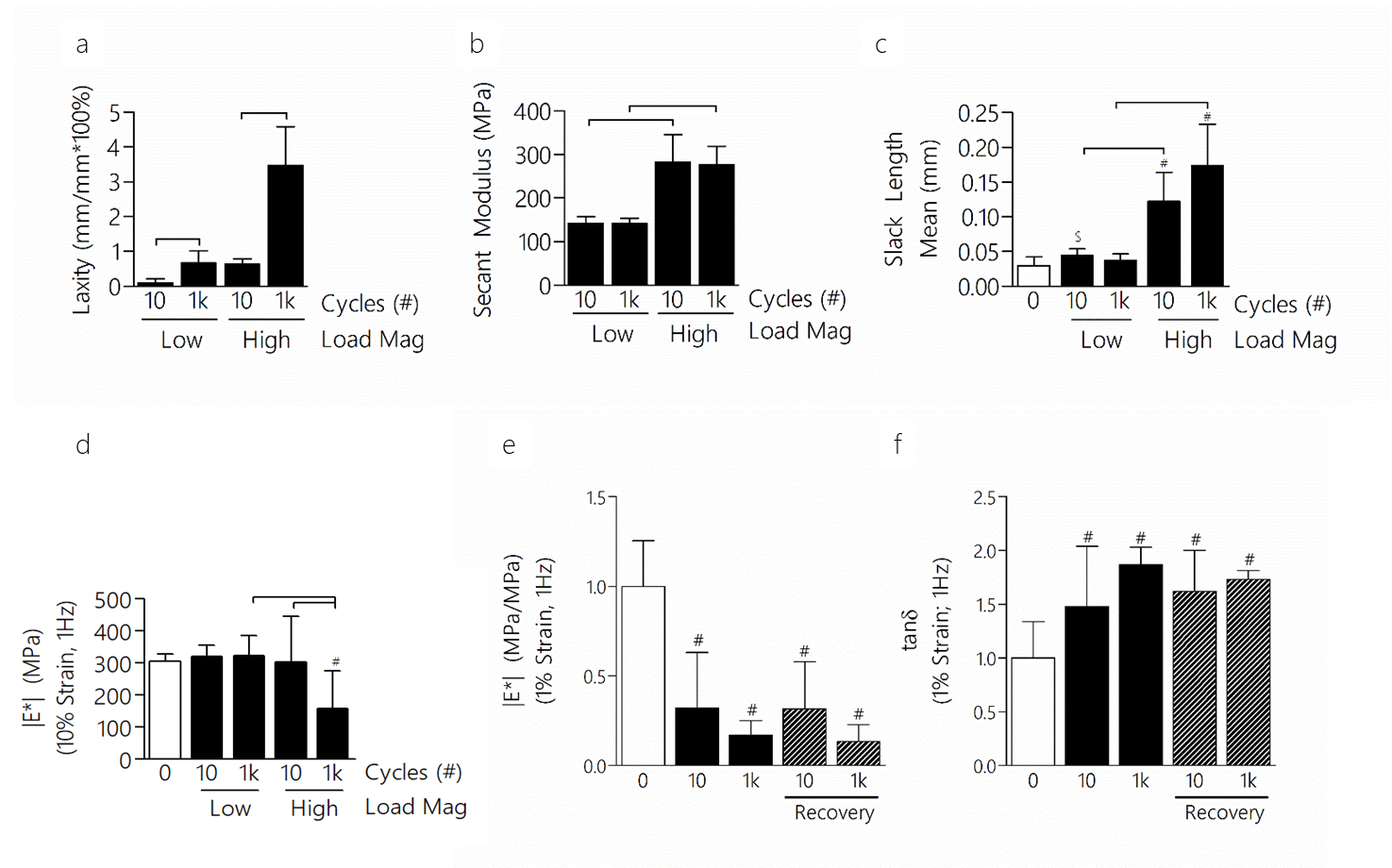
**

**Figure S4: Macro-mechanics with recovery: laxity, secant modulus in uninjured tendons. (A)** Cycle duration increased tendon laxity. (**B**) Loading magnitude represented the toe/transition and linear regions of the load-displacement curve as the detected secant modulus was lower in low magnitude loading groups. (**C**) The slack length increased as a result of high magnitude loading. (**D**) |E*| was decreased due to high/1k loading at 10% strain. (**E-F**) The mechanical properties |E*| and tanδ were affected by high magnitude loading and were non-recoverable. Data shown as mean ± SD. Symbols indicate significant differences (#) or trends ($) compared to quasi-static loaded samples. N=7-11/group. Lines indicate significant differences after Bonferroni correction.

**
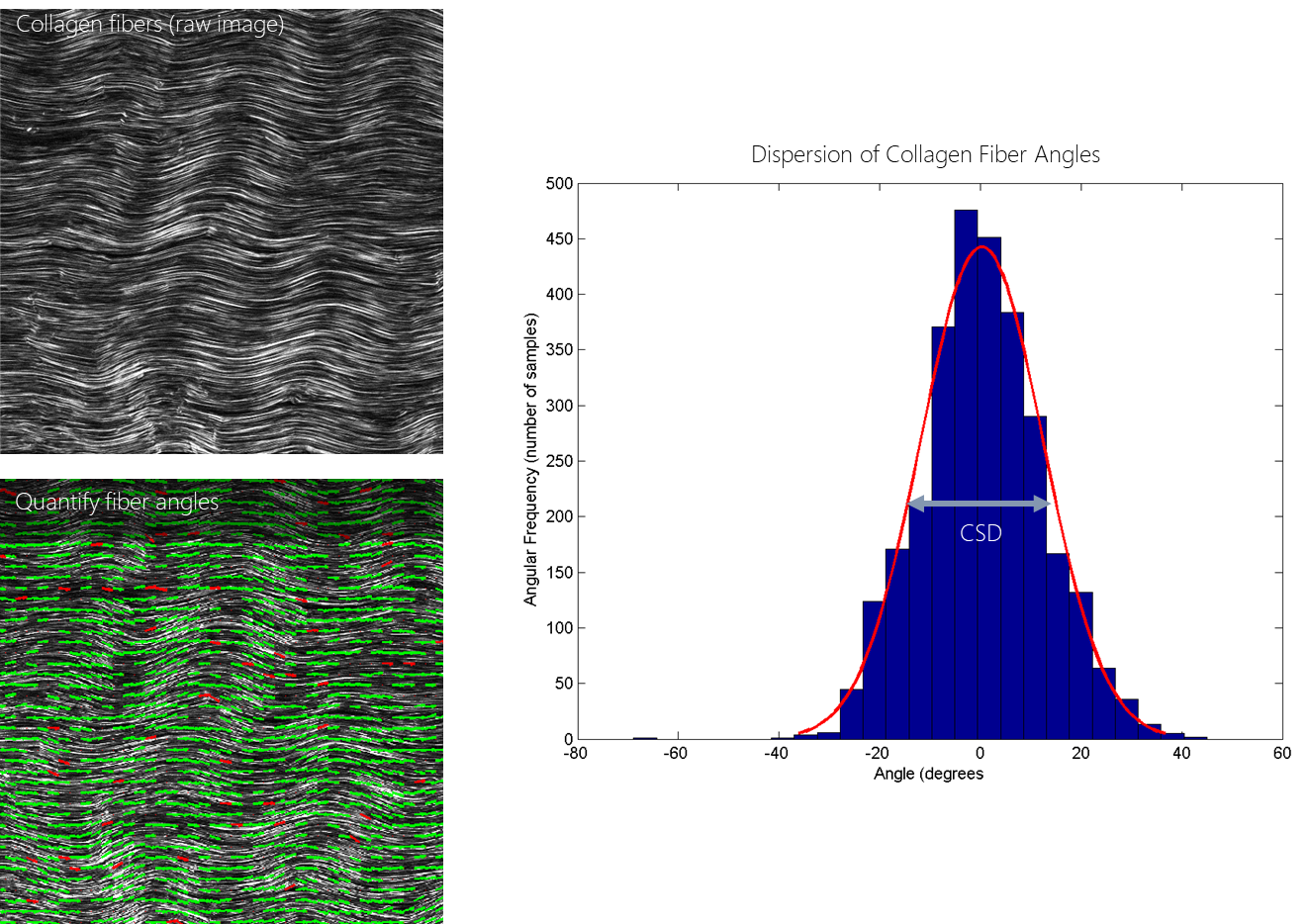
**

**Figure S5: Evaluation of collagen fiber disorganization.** A custom MATLAB program was used to evaluate collagen fiber organization from SHG images. The disorganization of collagen fibers was quantified by computing the circular standard deviation (CSD).

**
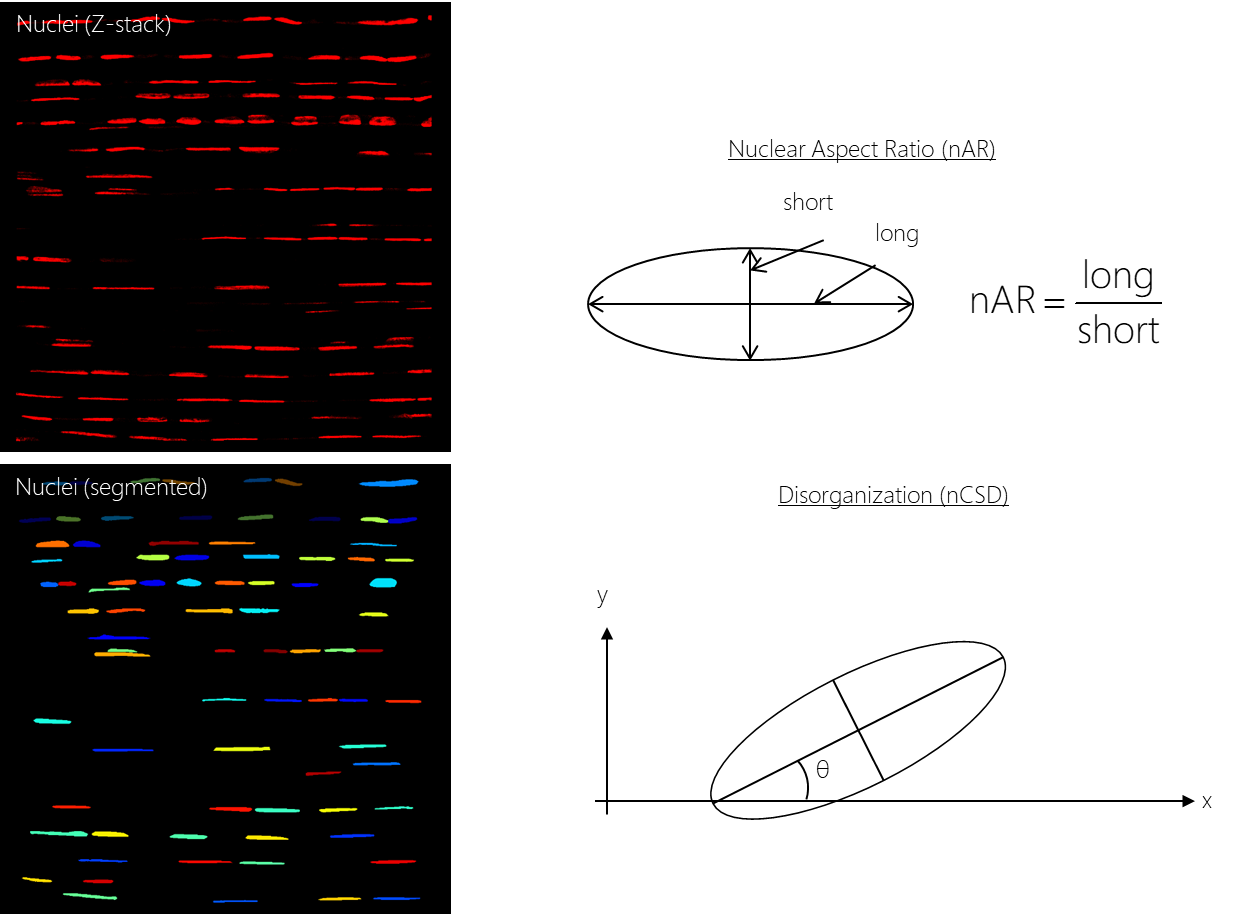
**

**Figure S6: Evaluation of nuclear aspect ratio and disorganization.** Nuclei were segmented and evaluated for their aspect ratio (nAR) defined by the ratio of the long and short axes. In addition, the disorganization of nuclei was evaluated (nCSD).

**
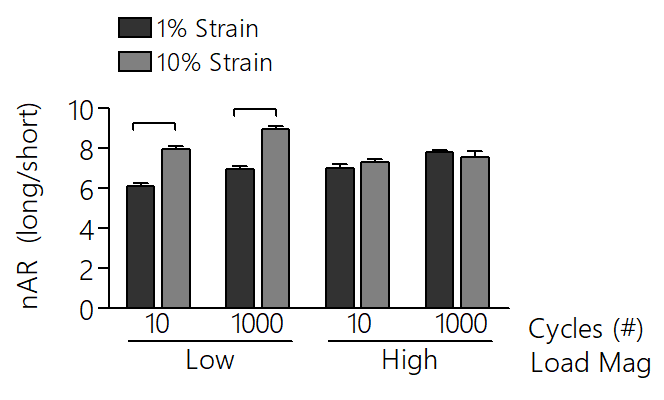
**

**Figure S7: Effect of loading on nAR.** The nAR increased from 1 to 10% strain following low cyclic loading, but did not change following high dynamic loading.


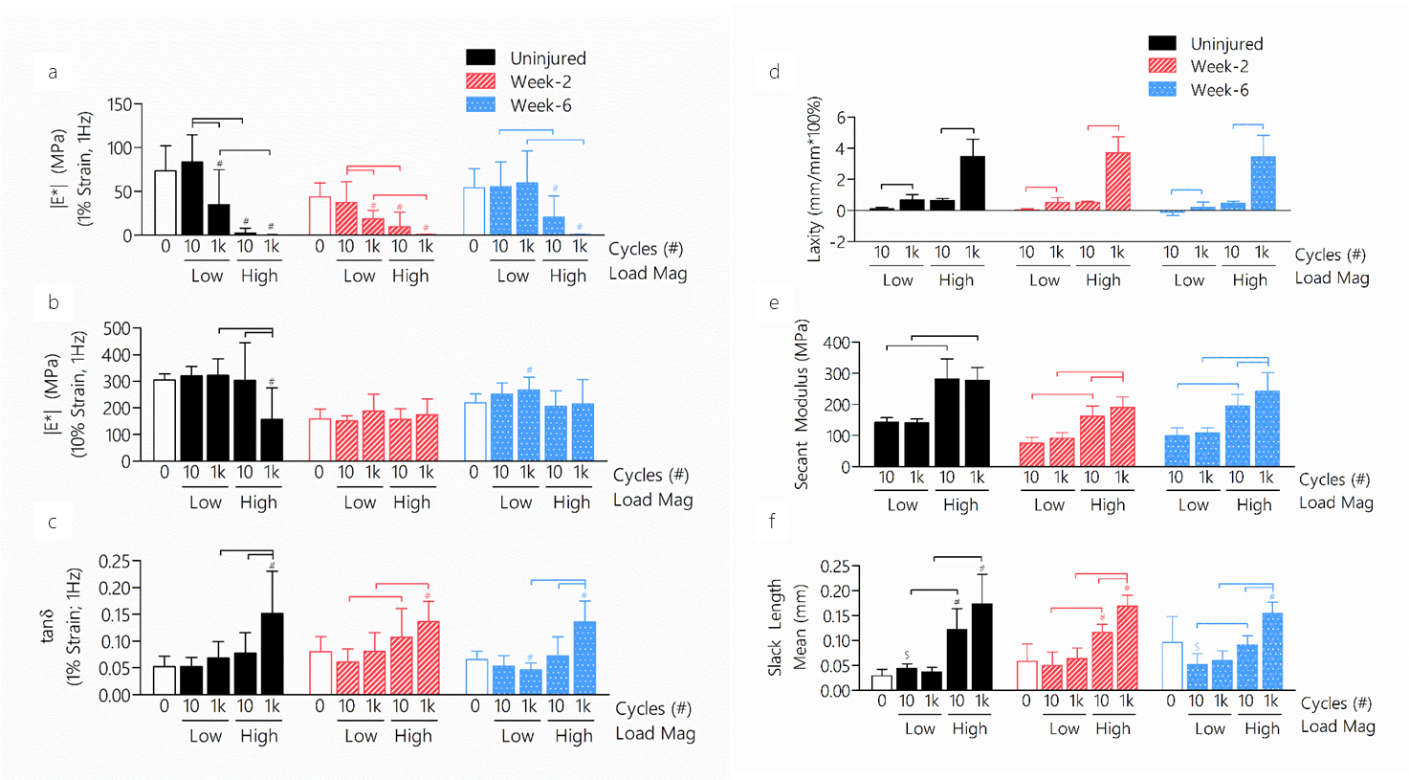


**Figure S8: Macroscale mechanics following dynamic loading and healing.** The effect of loading magnitude and cycle duration on (**A**) |E*| at 1% strain, (**B**) |E*| at 10% strain, (**C**) tanδ at 1% strain, (**D**) laxity, (**E**) secant modulus, and (**F**) fiber slack lengths depended on tendon healing. Data shown as mean±SD. N=7-11/group. Lines indicate significant differences after Bonferroni correction. Symbols indicate significant differences (#) or trends ($) compared to quasi-static loading samples (0 cycles).

**
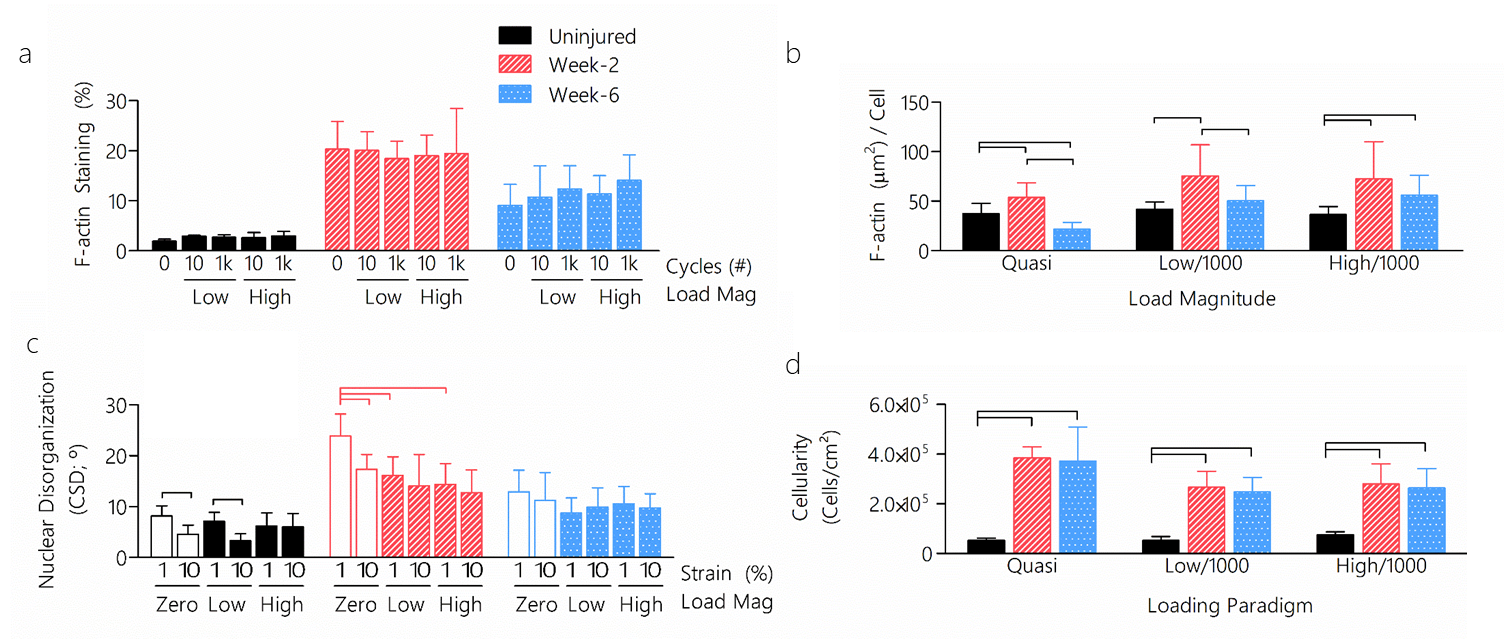
**

**Figure S9: Microscale actin staining and nuclear disorganization with healing.** (**A**) Loading magnitude and cycle duration did not affect F-actin staining, however, (**B**) tendon healing was a significant factor. (**C**) Nuclei reorganized with applied strain in uninjured tendons except after high magnitude loading. In contrast, nuclei in healing tendons did not reorganize with applied strain after dynamic loading. (**D**) Cellularity was increased in healing tendons. Data shown as mean±SD. N=7-11/group. Lines indicate significant differences after Bonferroni correction.

**
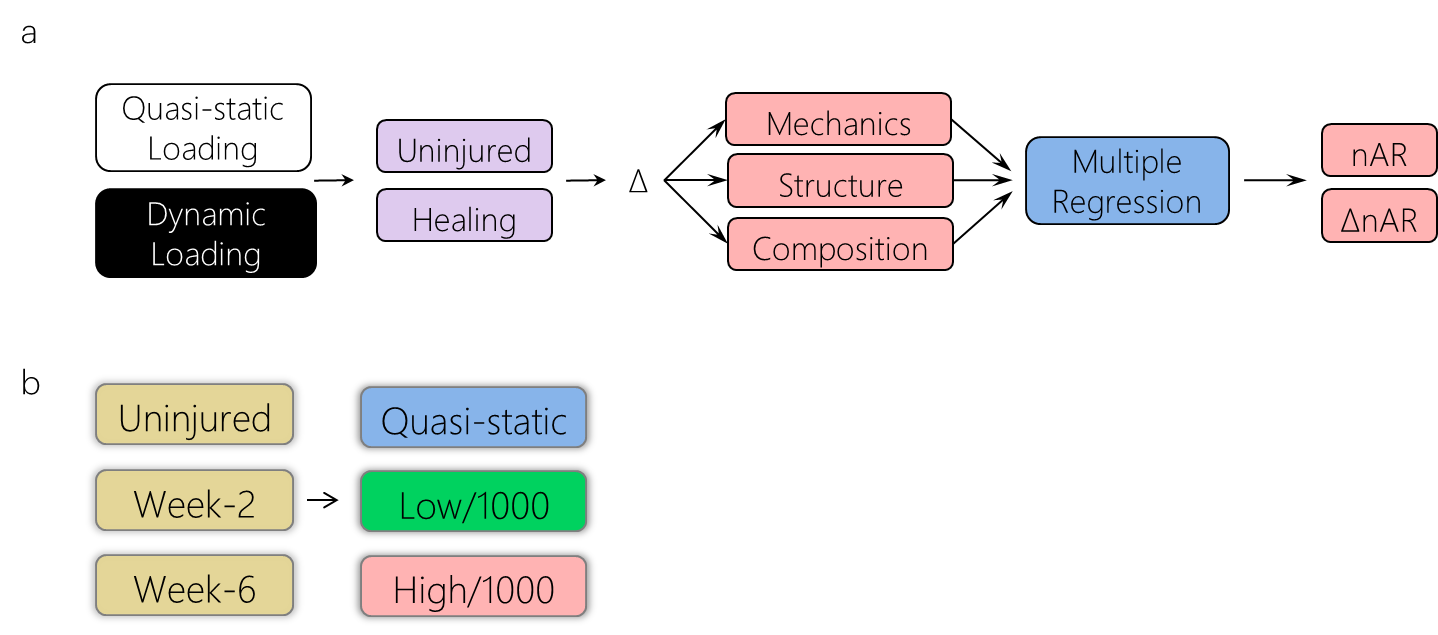
**

**Figure S10: Inputs for modeling framework.** (**A**) For multiple regression modeling, mechanical, structural, and compositional properties in uninjured and healing tendons acquired during quasi-static and dynamic loading were used to predict nAR and ΔnAR. (**B**) For modeling ECM stress transmission, uninjured, 2-week post-injury, and 6-week post-injury tendons tested during quasi-static, low/1000, and high/1000 loading were used (9 groups total).

**
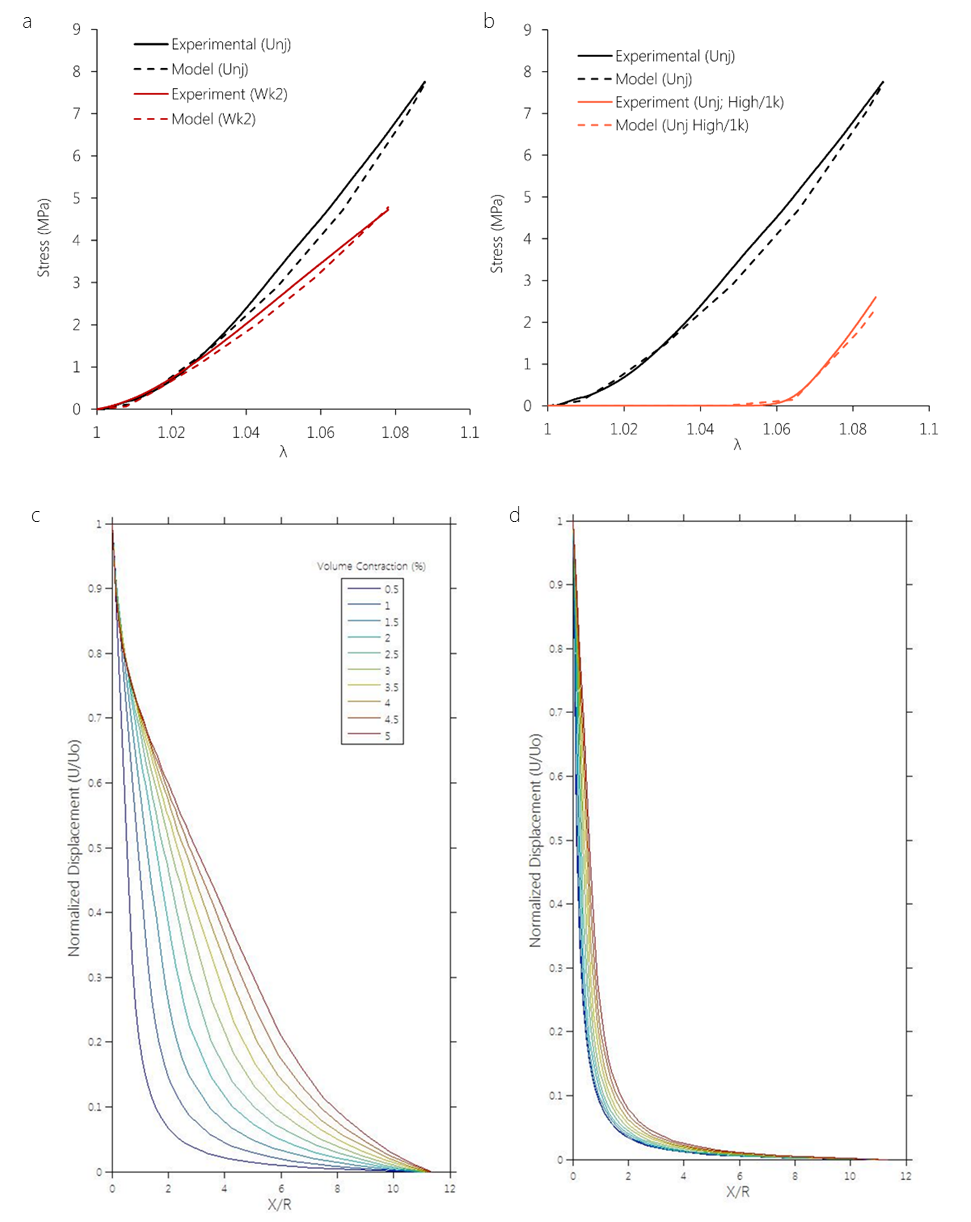
**

**Figure S11: Cell-generated stress transmission through the ECM: Model fits.** Model fits were determined for test conditions showing good agreement (for example) for (**A**) the effect of tendon healing, and (**B**) the effect of high magnitude loading. During cell contraction, ECM stresses were transmitted over long distances (up to 10x the cell length). ECM stress transmission was greater in (**C**) uninjured samples compared to those with (**D**) high magnitude loading.

**
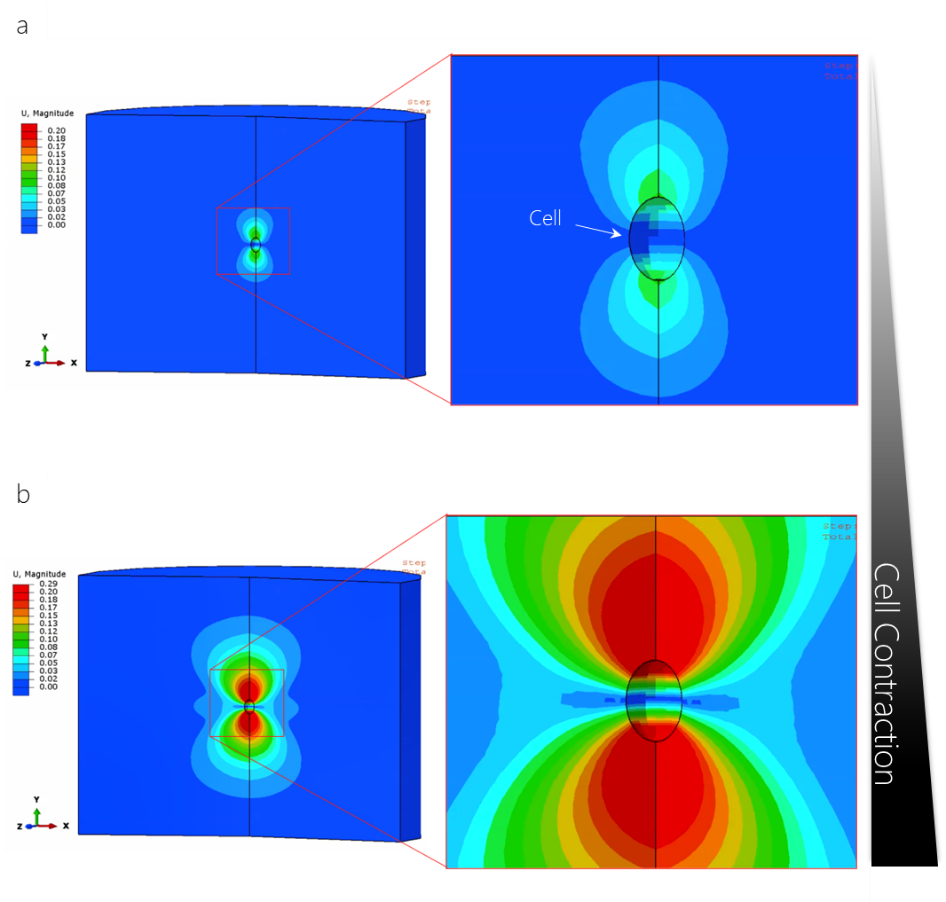
**

**Figure S12: Effect of cell contraction on ECM stress transmission.** (**A-B**) As the elliptical cell contracts along the vertical direction towards its centroid, forces are transmit from the cell border through the modeled ECM.
